# ITPR2 Mediated Calcium Homeostasis in Oligodendrocytes is Essential for Myelination and Involved in Depressive‐Like Behavior in Adolescent Mice

**DOI:** 10.1002/advs.202306498

**Published:** 2024-03-13

**Authors:** Ming Zhang, Na Zhi, Jiaxiang Feng, Yingqi Liu, Meixia Zhang, Dingxi Liu, Jie Yuan, Yuhao Dong, Sufang Jiang, Junye Ge, Shengxi Wu, Xianghui Zhao

**Affiliations:** ^1^ Department of Neuroscience Air Force Medical University Xi'an 710032 P. R. China; ^2^ College of Life Sciences Northwest University Xi'an 710127 P. R. China; ^3^ School of Life Science and Technology Xi'an Jiaotong University Xi'an 710049 P. R. China; ^4^ First Affiliated Hospital of Medical College Xi'an Jiaotong University Xi'an 710061 P. R. China

**Keywords:** calcium homeostasis, depressive‐like behaviors, ITPR2, oligodendrocytes

## Abstract

Ca^2+^ signaling is essential for oligodendrocyte (OL) development and myelin formation. Inositol 1,4,5‐trisphosphate receptor type 2 (ITPR2) is an endoplasmic reticulum calcium channel and shows stage‐dependent high levels in postmitotic oligodendrocyte precursor cells (OPCs). The role and potential mechanism of ITPR2 in OLs remain unclear. In this study, it is revealed that loss of *Itpr2* in OLs disturbs Ca^2+^ homeostasis and inhibits myelination in adolescent mice. Animals with OL‐specific deletion of *Itpr2* exhibit anxiety/depressive‐like behaviors and manifest with interrupted OPC proliferation, leading to fewer mature OLs in the brain. Detailed transcriptome profiling and signal pathway analysis suggest that MAPK/ERK‐CDK6/cyclin D1 axis underlies the interfered cell cycle progression in *Itpr2* ablated OPCs. Besides, blocking MAPK/ERK pathway significantly improves the delayed OPC differentiation and myelination in *Itpr2* mutant. Notably, the resting [Ca^2+^]_i_ is increased in *Itpr2* ablated OPCs, with the elevation of several plasma calcium channels. Antagonists against these plasma calcium channels can normalize the resting [Ca^2+^]_i_ level and enhance lineage progression in *Itpr2‐*ablated OPCs. Together, the findings reveal novel insights for calcium homeostasis in manipulating developmental transition from OPCs to pre‐OLs; additionally, the involvement of OLs‐originated ITPR2 in depressive behaviors provides new therapeutic strategies to alleviate myelin‐associated psychiatric disorders.

## Graphical abstract

ITPR2‐mediated calcium homeostasis is crucial for OPC development and the underlying molecular machinery changes following *Itpr2* depletion will finally interfere with OPC proliferation/differentiation progression and result in abnormal behaviors in the animal.

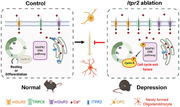

## 1 Introduction

Myelin, produced by oligodendrocytes (OLs), supports the rapid and synchronized transduction of information across the brain.^[^ ^1^ ^]^ The maturation of oligodendrocyte precursor cells (OPCs) into myelinating OLs is a highly regulated process and its disruption has been reported in many neuropsychiatric disorders, such as depression, autism, and schizophrenia.^[^ ^2^ ^]^ Clinical evidence from postmortem brain tissue, multimodal MR imaging, and single‐nucleus RNA‐Seq study has implicated myelin abnormalities in major depressive disorders.^[^ ^3^ ^]^ Studies support that drug mediated enhancement of OPC differentiation and myelination could rescue the depressive‐like behavior in demyelinating mice model, suggesting OLs as one of the potential treatment targets for these diseases.^[^ ^4^ ^]^

OL lineage cells express a wide range of Ca^2+^ channels or receptors that regulate intracellular Ca^2+^ levels,^[^ ^5^ ^]^ and Ca^2+^ signaling is an essential modulator for OPC development and myelin formation.^[^ ^5^ ^]^ For instance, intracellular calcium concentrations ([Ca^2+^]_i_) in a specific subset of OPCs mediate activity‐regulated cell proliferation.^[^ ^6^ ^]^ In our previous research, we found that increasing internal calcium release or extracellular calcium influx can rescue the differentiation defects of OPCs in vitro.^[^ ^7^ ^]^ Besides, we observed that ablation of endoplasmic reticulum (ER) located calcium channel, IP3R2 (also known as ITPR2), in the OL lineage significantly impairs OPC differentiation^[^ ^7^ ^]^ and the underlying signaling mechanisms remains elusive.

Although there are multiple correlation studies for psychiatric disorders and serum calcium levels,^[^ ^8^ ^]^ the association between intracellular calcium homeostasis in neuronal cells and depression is yet to be determined. In current study, by using *Itpr2* conditional knockout mice (*Itpr2* cKO) from our previous study,^[^ ^7^ ^]^ and generating another inducible *Itpr2* conditional knockout mice line (*Itpr2* iKO), we confirmed that ITPR2 is required for proper myelination and its depletion in OLs induced anxiety/depression‐like behaviors in the animals. Notably, we found that ablation of *Itpr2* may increase the resting [Ca^2+^]_i_ through compensatory elevation of cell membrane Ca^2+^ channel or receptors, which then activates MAPK/ERK pathway. Besides, cell cycle regulator cyclin D1 was activated in response to ERK activation and provided possible explanations for delayed cell cycle exit and OPC differentiation. Finally, antagonist against ERK pathway rescued hypomyelination and depressive like behaviors in *Itpr2* cKO mice. These results suggest the significance of ITPR2 mediated calcium homeostasis in oligodendrocytes and provide novel strategies for treating dysmyelination associated diseases.

## 2 Results

### 2.1 Loss of *Itpr2* in OLs Disturbs Ca^2+^ Release from ER

**Figure 1:**
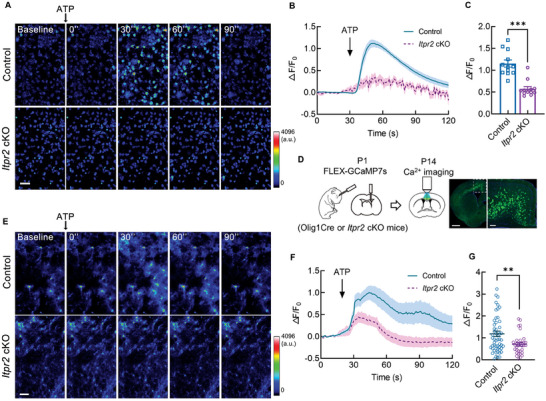
Lack of *Itpr2* impairs calcium release from ER in OPCs. A) Representative serial heatmap of calcium imaging for living OPC cultures after the addition of 100 µm ATP, as revealed by Fluo‐8 AM. Cultures from Olig1Cre mice were used as control. Bar = 50 µm. B) Representative trace of Ca^2+^ release indicated by Fluo‐8 fluoresces intensity in OPCs following addition of ATP (*n* = 50 traces for control and cKO group from 3 individual cell cultures, respectively). C) Amplitude of relative [Ca^2+^]_i_ changes after ATP treatment in *Itpr2* cKO and control OPCs (*n* = 12 independent cultured coverslips). Data presents Mean ± SEM; Mann Whitney test, ***, *p* <0.001. D) Schematic diagram of virus injection in lateral ventricle of P1 pups and calcium imaging for brain slice including anterior cingulate cortex from P14 mice. Representative image showing the position of GCaMP7s‐GFP expression was on the right. Bar = 1 mm (left) and 200 µm (right). E) Representative serial heatmap of calcium imaging of brain slices labeled with rAAV2/9‐CAG‐FLEX‐GCaMP7s from *Itpr2* cKO and control (Olig1Cre) mice, Bar = 50 µm. F) Representative trace of GCaMP7s intensity following ATP application (*n* = 30 traces for control and cKO group from 3 mice, respectively). G) Amplitude of relative [Ca^2+^]_i_ changes after ATP treatment in *Itpr2* cKO and control mice (*n* = 50 cells for control and cKO group from 3 mice). Data presents Mean ± SEM; Unpaired *t*‐test with Welch's correction, **, *p* <0.01.

As an endoplasmic reticulum (ER) calcium channel, ITPR2 can be triggered by inositol 1,4,5‐trisphosphate (IP3) elevation and increases the [Ca^2+^]_i_.^[^ ^5^ ^]^ To confirm the effect of ITPR2 ablation on Ca^2+^ release from ER in OPCs, we performed Ca^2+^ imaging with calcium indicator Fluo‐8 AM as introduced in previous studies.^[^ ^7^, ^9^ ^]^ All loading and imaging process were performed in calcium free artificial cerebrospinal fluid (ACSF) to exclude possible Ca^2+^ influx. ATP can initiate IP3 production through a group of G‐protein‐coupled receptors, including purinergic P2Y2 receptor, then we stimulated purified OPC cultures from *Itpr2* cKO mice^[^ ^7^ ^]^ with ATP. Compared to the transient and strong increase of calcium waves in control group (from Olig1Cre mice), we observed significantly reduced calcium fluctuation in *Itpr2* cKO group (**Figure** **1**A–C). To further confirm the impaired calcium reflection in vivo, rAAV2/9‐CAG‐FLEX‐GCaMP7s were injected into intracerebroventricular of postnatal day 1 (P1) mice and used to reveal calcium transients in oligodendrocytes. Immunostaining the brain slices expressing GCaMP7s‐GFP revealed that GFP labeled cells were almost all positive for SOX10 (98.97±1.193%), but not for GFAP in P14 control cortex (Figure S1A,B, Supporting Information), indicating the oligodendrocyte lineage specificity of in vivo calcium imaging. Besides, OL stage specific marker staining showed that 58.41±2.160% GFP labeled cells were positive for PDGFRα, 11.50±1.330% GFP cells were O4^+^ immature OL and 23.31±3.109% GFP cells were CC1^+^ mature OL (Figure S1C–G, Supporting Information). This result indicated that calcium transients recorded by in vivo calcium imaging were mainly from OPCs. Calcium imaging was then performed on acutely isolated brain slices including prefrontal cortex from P14 control and cKO mice (Figure 1D). Similar to cell culture results, ATP application induced a robust Ca^2+^ release in oligodendrocytes from control brain slice, as indicated by a simultaneous increase in GFP intensity, but Ca^2+^ signal changes in cKO group were significantly reduced (Figure 1E–G). These results suggest that *Itpr2* is essential for calcium activity in oligodendrocytes and we then assessed the consequences of its loss in these cells.

### 2.2 *Itpr2* Ablation in OLs Results in Hypomyelination in the Brain

In an early single‐cell sequencing study for the brain, ITPR2 showed the highest expression in postmitotic newly formed OLs.^[^ ^10^ ^]^ We confirmed its expression pattern with immunostaining during early developmental stage. As revealed in Figure S2A‐B (Supporting Information), *Itpr2* was restrictedly expressed in SOX10 labeled OL lineage cells in the corpus callosum (cc) and showed highest level in P14 mice, at which stage the number of newly formed OLs reached the peak. According to our previous study, conditional ablation of *Itpr2* from pre‐OPC stage results in hypomyelination in adolescent mice.^[^ ^7^ ^]^ To further confirm the role of ITPR2 in OPCs after birth, we bred *Itpr2* ^fl/fl^ mice with NG2‐Cre^ER^ line, an OPC‐specific inducible Cre line to generate *Itpr2* ^fl/fl^; NG2‐Cre^ER^ (*Itpr2* iKO) mice. Tamoxifen was intraperitoneal injected daily into *Itpr2* iKO mice from P3‐P6 to induce *Itpr2* deletion (Figure S2C, Supporting Information), and double immunostaining confirmed ITPR2 loss in SOX10^+^ OLs (Figure S2D,E, Supporting Information). *Itpr2* ^fl/fl^ mice injected with Tamoxifen were served as control, since tamoxifen has been reported to promote differentiation of OPCs in vitro^[^ ^11^ ^]^ and accelerate the repair of demyelination lesions in the CNS.^[^ ^12^ ^]^

**Figure 2:**
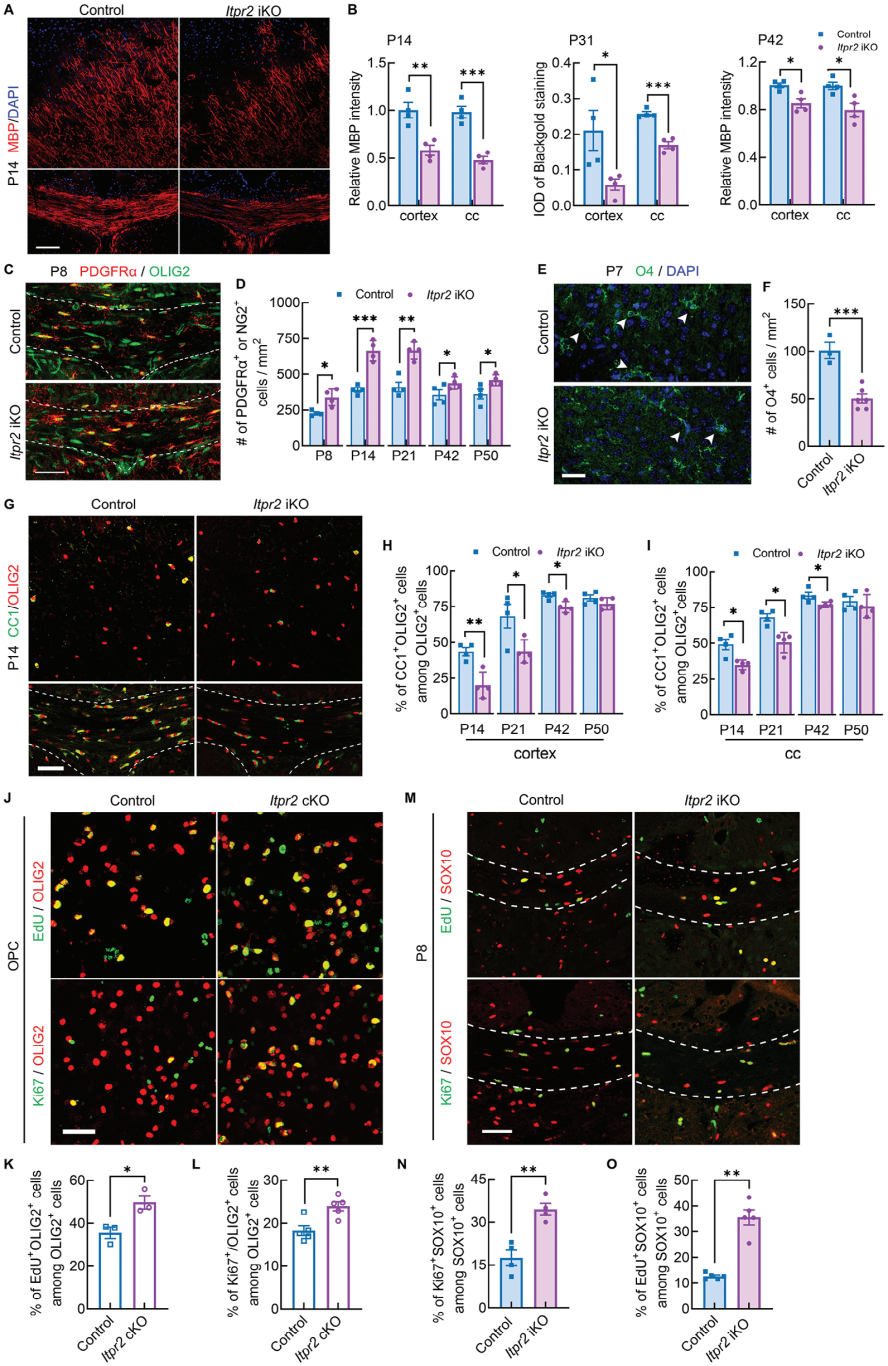
ITPR2 is required for OL maturation and proper proliferation of OPCs. A) Representative MBP immunostaining in cortex and corpus callosum of control and iKO mice at P14. Bar = 200 µm. B) Quantification of MBP intensity or black gold staining in cortex and cc of control and iKO mice at P14, P31 and P42 (*n* = 4 animals each group). Data presents Mean ± SEM; Two tail unpaired *t‐*test, *, *p* <0.05; **, *p* < 0.01; ***, *p* <0.001. C) Representative immunostaining for PDGFRα and OLIG2 in corpus callosum from control and iKO mice at P8. Bar = 50 µm. D) Quantification the density of PDGFRα^+^ or NG2^+^ cells in corpus callosum from control and iKO mice at P8, P14, P21, P42 and P50 (*n* = 4 mice for control and iKO group). Data presents Mean ± SEM; Two tail unpaired *t*‐test, *, *p* <0.05; **, *p* < 0.01; ***, *p* < 0.001. E) Representative immunostaining for O4 in cortex from control and iKO mice at P7. Bar = 50 µm. F) Quantification the density of O4^+^ cells in cortex from control and iKO mice at P7 (*n* = 4 animals for each group). Data presents Mean ± SEM; Two tail unpaired *t*‐test, ***, *p* < 0.001. G) Representative immunostaining for CC1 and OLIG2 to identify mature OL in cortex and corpus callosum from P14 control and iKO mice. Bar = 50 µm. H) Quantification the proportion of CC1^+^ mature OLs among OLIG2^+^ cells in cortex from control and iKO mice at P14, P21, P42 and P50 (*n* = 4 animals for each group). Data presents Mean ± SEM; Two tail unpaired *t*‐test, *, *p* <0.05; **, *p* < 0.01. I) Quantification the proportion of CC1^+^ mature OLs among OLIG2^+^ cells in corpus callosum from control and iKO mice at P14, P21, P42 and P50 (*n* = 4 animals for each group). Data presents Mean ± SEM; Two tail unpaired *t*‐test, *, *p* <0.05. J) Immunostaining of OLIG2 with EdU or Ki67 in purified OPC cultures from control and cKO mice. Bar = 50 µm. K) Percentage of EdU^+^ cells among OLIG2^+^ cells (*n* = 3 slides from 3 mice for each group). Data presents Mean ± SEM; Two tail unpaired *t*‐test; *, *p* <0.05. L) Percentage of Ki67^+^ cells among OLIG2^+^ cells (*n* = 5 slides from 3 mice for each group). Data presents Mean ± SEM; Two tail unpaired *t* test; **, *p* <0.01. M) Immunostaining for SOX10 with EdU and Ki67 to identify proliferating OPCs in corpus callosum from control and iKO mice at P8. Bar = 50 µm. N) Quantification the proportion of Ki67^+^SOX10^+^ proliferating OPCs in corpus callosum (*n* = 4 mice for each group). Data presents Mean ± SEM; Two tail unpaired *t*‐test; **, *p* <0.01. O) Quantification the proportion of EdU^+^SOX10^+^ cells in corpus callosum (*n* = 5 mice for each group). Data presents Mean ± SEM; Two tail unpaired *t*‐test; **, *p* <0.01.

We accessed the myelin structure by Black gold II staining or MBP immunostaining at P14, P31, and P42 mice (**Figure** **2**A; Figure S3A,B, Supporting Information). The integrated optical density (IOD) of myelin staining from cortex and cc was significantly decreased in iKO mice at all stages (Figure 2B). The comparable NEFL2 staining between groups excluded the possible axonal degeneration in iKO mice (Figure S2C,D, Supporting Information). In addition, electron microscopy (EM) revealed that the number of myelinated axons in the corpus callosum was significantly reduced in iKO mice compared to controls at P27 and P42 (Figure S3E,F,H, Supporting Information). Moreover, those myelinated axons in iKO mice were characterized by higher G ratios and thinner myelin sheaths than those of control mice (Figure S3E,G,I, Supporting Information). For P50 adult mice, although the number of myelinated axons in iKO mice was comparable to control mice, the thickness of the myelin sheath remained reduced (Figure S3E,J,K, Supporting Information). Together, these observations suggest a stage‐dependent function of ITPR2 in CNS myelination.

To test whether hypomyelination in *Itpr2* iKO mice was induced by increased apoptosis and/or arrested differentiation in OLs, we performed TUNEL assay and immunostaining for OL stage specific marker during development, respectively. In the brain of P7 iKO mice, we did not detect a significant increase of apoptotic OLs (Figure S4A,B, Supporting Information). In contrast, we observed a slight but remarkable expansion of PDGFRα^+^ or NG2^+^ OPCs from P8 to P50 in the corpus callosum of *Itpr2* iKO mice (Figure 2C,D; Figure S4C, Supporting Information), with reduced OLIG2^+^ cells in P8 and comparable amount of OLIG2^+^ OL linage cells after P14 (Figure S4D, Supporting Information). In addition, the density of O4^+^ pre‐OLs in the P7 cortex and the percentage of CC1^+^ mature OLs among OLIG2^+^ cells in the P14 cortex was significantly reduced in *Itpr2* iKO mice (Figure 2E‐H). This reduction was reproduced in P21 and P42 mice and recovered in P50 (Figure 2H), at which stage the density of OLIG2^+^ OL linage cells is comparable between groups in the cortex (Figure S4E, Supporting Information). Similar results were revealed in the corpus callosum from different developmental stages (Figure 2G,I). Together, these data suggest that *Itpr2* ablation may delay OPC differentiation by disturbing cellular proliferation.

The linkages between OPC proliferation and Ca^2+^ homeostasis have been previously reported. For example, overexpression of OPC‐specific calcium‐permeable AMPA receptors, GluA2, significantly increased the proliferation of OPC after demyelinating brain injury.^[^ ^13^ ^]^ To verify the role of ITPR2 in OPC proliferation, we first performed EdU incorporation assay in OPC cultures. Immunostaining revealed a significant increase in the percentage of EdU^+^ cells among OLIG2^+^ cells in *Itpr2* cKO group (Figure 2J‐K). Besides, another proliferation marker, Ki67, showed a higher percentage among OLIG2^+^ cells in OPC cultures from *Itpr2* cKO mice (Figure 2J,L). Similar Ki67 staining results were obtained in corpus callosum from P8 *Itpr2* iKO mice brain (Figure 2M,N). To examine the role of ITPR2 in regulating the transition from OPCs to OLs, EdU incorporation assay was performed in *Itpr2* iKO mice that were intraperitoneally injected with EdU at P6 and collected for analysis at P8. We noticed that the percentage of EdU^+^ cells among SOX10^+^ cells was greatly increased in *Itpr2* iKO mice (Figure 2M,O), and these EdU^+^ cells showed less co‐immunostaining with mature OL marker CC1 and more co‐staining with PDGFRα^+^ OPCs in *Itpr2* iKO mice (Figure S5, Supporting Information). These data suggest that ITPR2 loss in oligodendrocytes may keep these cells in OPC stage, rather than transiting into differentiation. Consistently, the comparable total number of oligodendrocytes in *Itpr2* mutant may indicate the extended cell cycle in OPCs.

Together with the above‐stage marker staining result, we believe that the absence of *Itpr2* may inhibit OPC differentiation by interfering with the transition from OPC to the pre‐OL stage, which is highly consistent with the dynamic expression pattern of ITPR2 during OPC development.

### 2.3 *Itpr2* Loss in Oligodendrocytes Leads to Depressive‐Like Behaviors

**Figure 3:**
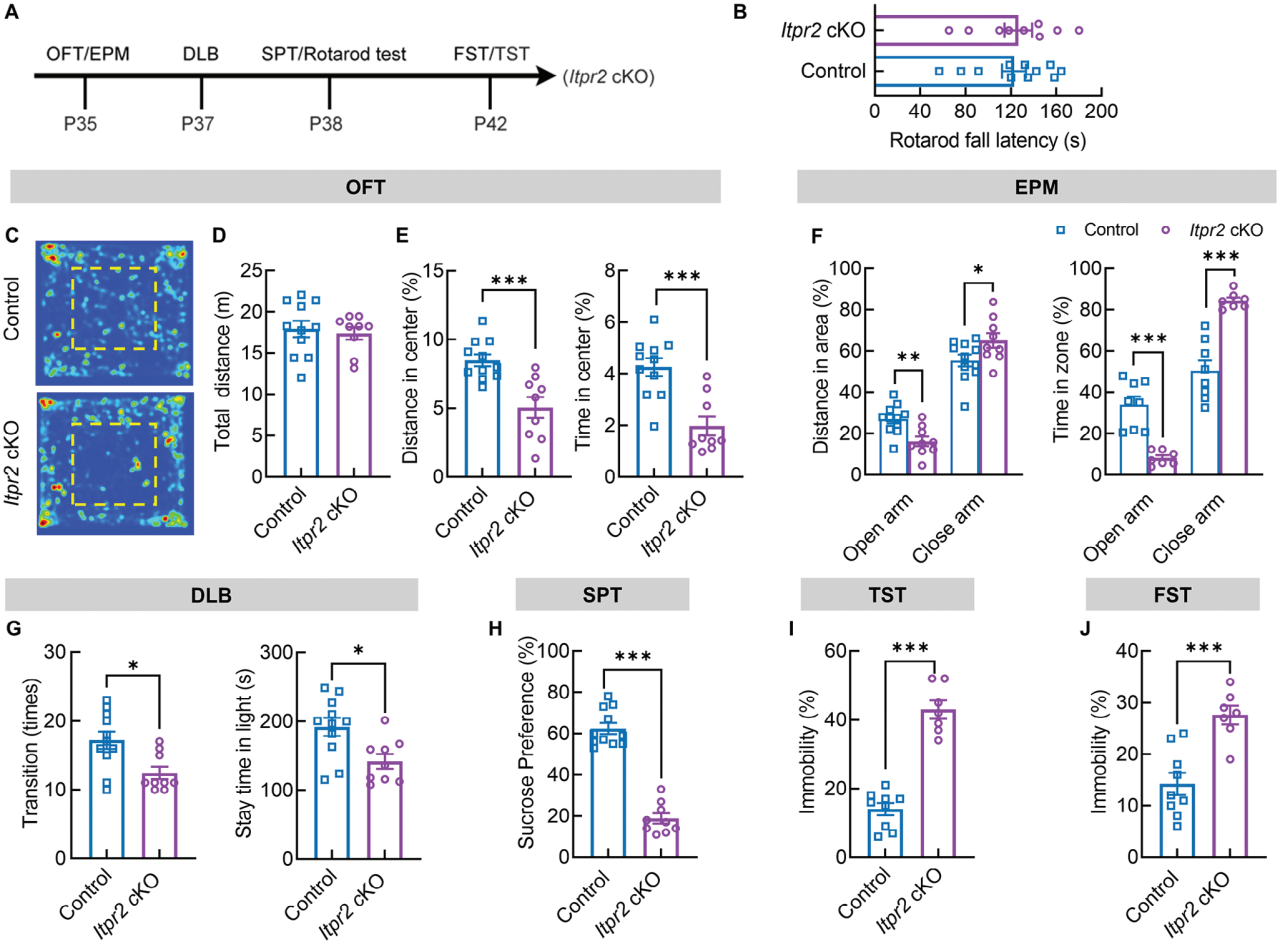
Increased depression/anxiety level in *Itpr2* cKO mice. A) Schematic diagram for mice behavioral test plan. OFT, open field test; EPM, elevated plus maze; DLB, dark light box; SPT, sucrose preference test; FST, forced swim test; TST, tail suspension test. B) Comparison of the fall latency in rotarod test between control and *Itpr2* cKO mice (*n* = 11 control mice and *n* = 9 cKO mice). Data presents Mean ± SEM; Two tail unpaired *t*‐test. C–E) *Itpr2* cKO mice revealed decreased activity in the central area of the open field. Representative heat maps of movements in the OFT (C). Comparison of total traveling distance during a 10‐min test between control and cKO mice (D). Percentage of traveling distance and time in the center zone of open field (E). (*n* = 11 control mice and *n* = 9 cKO mice). Data presents Mean ± SEM; Two tail unpaired *t*‐test and Mann–Whitney test; ***, *p* <0.001. F) *Itpr2* cKO mice revealed decreased activity in the open arm of EPM. Percentage of traveling distance (left) and time (right) in open arm and close arm of EPM. (*n* = 11 control mice and *n* = 9 cKO mice). Data presents Mean ± SEM; Two tail unpaired *t*‐test; *, *p* < 0.05; **, *p* <0.01; ***, *p* <0.001. G) *Itpr2* cKO mice showed fewer transition times (left) and less stay time in the lightbox (right). (*n* = 11 control mice and *n* = 9 cKO mice). Data presents Mean ± SEM; Two tail unpaired *t*‐test; ^*^ *p* <0.05. H) Percentage of sucrose consumption volume in SPT (*n* = 11 control mice and *n* = 9 cKO mice). Data presents Mean ± SEM; Two tail unpaired *t*‐test, ***, *p* <0.001. I) Percentage of immobility time in TST (*n* = 9 control mice and *n* = 7 cKO mice). Data presents Mean ± SEM; Two tail unpaired *t*‐test, ***, *p* <0.001. J) Percentage of immobility time in FST (*n* = 9 control mice and *n* = 7 cKO mice). Data presents Mean ± SEM; Two tail unpaired *t*‐test; ***, *p* <0.001.

Apart from neuronal disturbances, increasing evidence has pointed to oligodendrocyte dysfunction and demyelination in depressive disorder.^[^ ^3^, ^14^ ^]^ A single nucleus transcriptomic analysis for the prefrontal cortex from patients with major depressive disorders revealed that the most notable dysregulation occurred in excitatory neurons and immature OPCs,^[^ ^3c^ ^]^ indicating the significance of oligodendrocytes in this disease. Since *Itpr2* ablation in OLs results in hypomyelination and impaired OPC differentiation, we performed a series of behavioral tests to examine how these changes affected animal behaviors (**Figure** **3**A). The mutant did not reveal tremble and ataxia behavior as previously reported in several demyelination mice.^[^ ^15^ ^]^ To examine the motor coordination ability of *Itpr2* cKO mice, rotarod tests were applied to P38 mice. Under continuous accelerating rotating rod, *Itpr2* mutant showed comparable falling latency as control mice, indicating their normal motor ability (Figure 3B). In an open field test (OFT), *Itpr2* cKO mice at P35 traveled a similar total distance with control mice during a 10‐min test. However, they spent less time and traveled less distance in the central zone compared to control mice (Figure 3C–E). Similarly, cKO mice at P35 showed less activity in open arms and higher percentage of travel distance in closed arms in elevated plus maze (EPM) (Figure 3F). These observations indicate increased anxious level in *Itpr2* mutant. We then confirmed the results in another anxiety‐related test, dark–light box (DLB) transition test. *Itpr2* cKO mice at P37 exhibited less transitions between dark and light box, and their stay duration in the light area was significantly decreased compared to control mice (Figure 3G).

As patients with anxiety are often accompanied by depression,^[^ ^16^ ^]^ we then examined the depressive like behaviors in *Itpr2* cKO mice. As shown in Figure 3H, *Itpr2* cKO mice at P38 showed less sucrose consumption than control mice in the sucrose preference test (SPT). And their immobility time in the tail suspension test (TST) was significantly increased compared with the control mice at P42 (Figure 3I). Besides, in the forced swimming test (FST), *Itpr2* cKO mice at P42 showed less desire for struggling in the water, and their immobility time was longer than the control (Figure 3J).

To further confirm these results, we tested the behavior changes in age‐matched *Itpr2* iKO mice (Figure S6A, Supporting Information). Similarly, iKO mice exhibited less exploration in the central open field and less active movement in the open arm of EPM (Figure S6B,C, Supporting Information). In the DLB test, they transited less times between the dark and light side and spent less time in the light box compared to control mice (Figure S6D, Supporting Information). Their immobility proportion during a 5‐min TST was significantly higher than control (Figure S6E, Supporting Information). Together, these results suggest that *Itpr2* loss in oligodendrocytes may induce anxiety and depressive‐like behaviors in the animals, thus strengthening the significance of OL calcium homeostasis in mediating neural functions.

### 2.4 *Itpr2* Ablation Changed Transcriptome Profiling in OPCs and Activated Cyclin D1 Through MAPK/ERK Pathway

**Figure 4:**
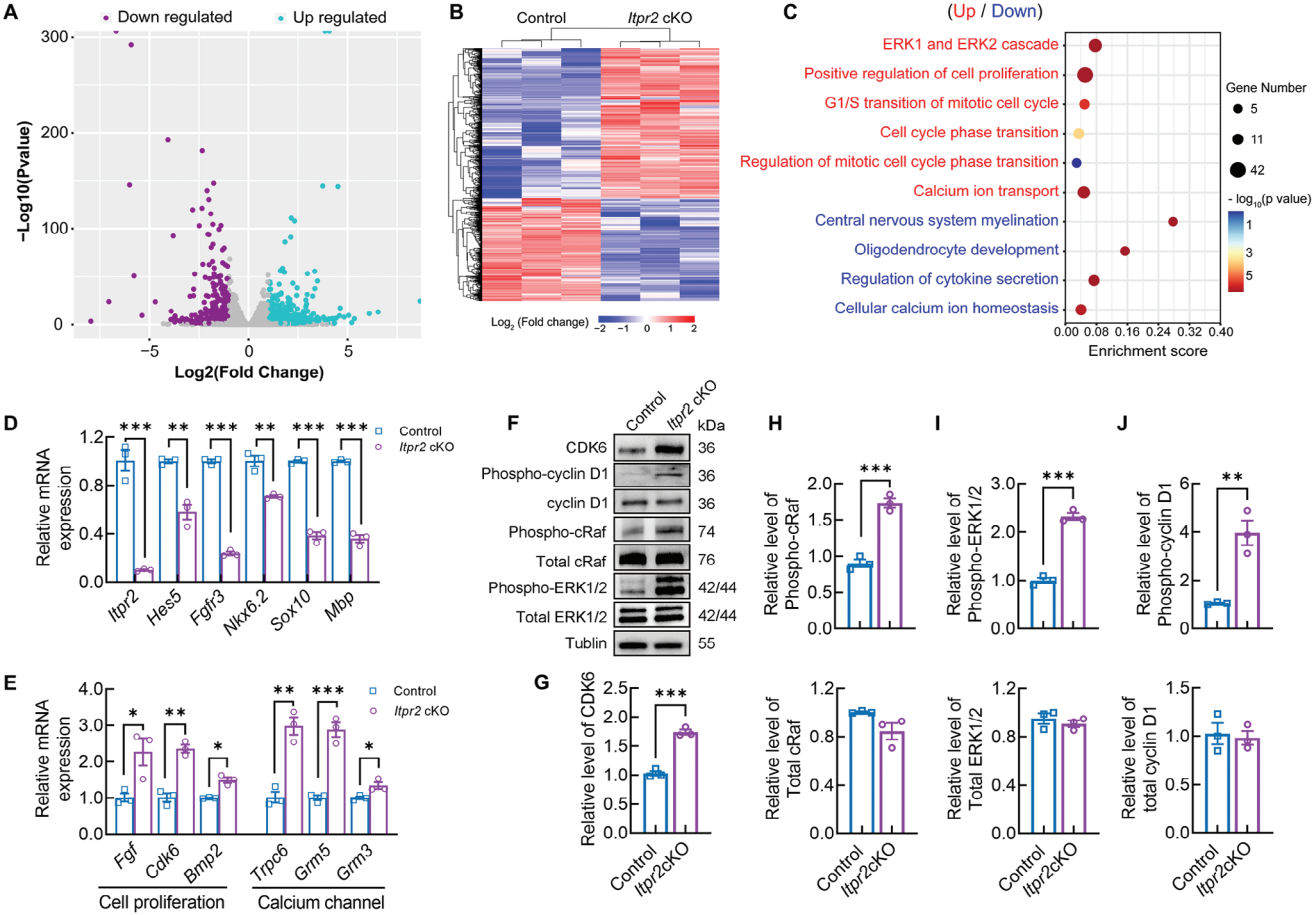
*Itpr2* ablation changed transcriptome profiling in OPCs. A) Volcano plot of genes with |log_2_ fold change| (> 1) on the X‐axis and log_10_ of the *p*‐value on the Y‐axis. Each point represents an individual gene; colored points represent significant DEGs, and grey points represent non‐significant genes. B) Hierarchical clustering of upregulated and downregulated genes in OPCs from cKO mice. *n* = 3 independent cell cultures per group. C) Bubble plots of representative functional clusters for DEGs from cKO mice. Red terms are from upregulated genes and blue terms are from downregulated genes. D) Validation of representative downregulated genes in *Itpr2* cKO OPCs by qRT‐PCR. (*n* = 3 independent experiments each performed in triplicate). Data presents Mean ± SEM of transcript levels relative to control after normalization; Two tail unpaired *t*‐test; **, *p* <0.01. ***, *p* <0.001. E) Validation of representative upregulated genes in *Itpr2* cKO OPCs by qRT‐PCR. (*n* = 3 independent experiments each performed in triplicate). Data presents Mean ± SEM of transcript levels relative to control after normalization; Two tail unpaired *t*‐test; *, *p* <0.05. **, *p* <0.01. ***, *p* <0.001. F) Western blot analysis showed increased levels of CDK6, Phospho‐cyclin D1, Phospho‐cRaf, and Phospho‐ERK1/2 in OPCs from cKO mice. Tubulin was used as the loading control. G) Relative CDK6 level in control and cKO OPCs (*n* = 3 mice for each group). Data presents Mean ± SEM; Two tail unpaired *t*‐test; ***, *p* <0.001. H) Relative phospho‐cRaf (upper) and total cRaf level (lower) in control and cKO OPCs (*n* = 3 mice for each group). Data presents Mean ± SEM; Two tail unpaired *t*‐test; ***, *p* <0.001. I) Relative phospho‐ERK1/2 (upper) and total ERK1/2 level (lower) in control and cKO OPCs (*n* = 3 mice for each group). Data presents Mean ± SEM; Two tail unpaired *t*‐test; ***, *p* <0.001. J) Relative phospho‐cyclin D1 (upper) and total cyclin D1 level (lower) in control and cKO OPCs (*n* = 3 mice for each group). Data presents Mean ± SEM; Two tail unpaired *t*‐test; **, *p* <0.01.

To systemically investigate the mechanism underlying the hypomyelination in the context of *Itpr2* deficiency, we performed RNA‐Seq analysis for primary OPC cultures from control and *Itpr2* cKO mice. *Itpr2* deficiency showed profound effects on the gene expression landscape, as hundreds of differentially expressed genes (DEGs) were detected (**Figure** **4**A,B). There are 577 downregulated and 840 upregulated genes in *Itpr2* cKO OPCs (|log_2_fold| ≥ 1; *p* value ≤ 0.05) (Figure 4A,B). Gene ontology (GO) analysis revealed that downregulated genes in *Itpr2* cKO OPCs were associated with myelination, oligodendrocyte development, and calcium ion homeostasis, while upregulated genes were linked to ERK1/2 cascade, cell proliferation, and mitotic cell cycle (Figure 4C). This is consistent with the aberrant cell proliferation and the hypomyelination phenotype in *Itpr2* cKO mice. We then confirmed the mRNA level changes of representative genes that related to OPC development and cell cycle regulation by qRT‐PCR assay (Figure 4D,E). Also, we noticed that a group of calcium channels is upregulated in the *Itpr2* cKO group, including the transient receptor potential cation channel (*Trpc6*) and metabotropic glutamate receptor channel (*Grm5*, *Grm3*) (Figure 4E). This may indicate the compensatory effects after *Itpr2* loss. Besides, we noticed the upregulation of cyclin‐dependent kinases 6 (CDK6) (Figure 4E), which is part of the G1 cell cycle checkpoint and allows the cell to enter the S‐phase.^[^ ^17^ ^]^ Its upregulation is highly consistent with more proliferating OPCs after *Itpr2* ablation. Therefore, it will be interesting to explore the underlying molecular networks after the break of ITPR2 mediated calcium homeostasis.

As one of the downstream effectors for calcium signaling, mitogen‐activated protein kinases (MAPK) cascades have been known to activate OPC proliferation during early development in the CNS^[^ ^18^ ^]^, and inhibiting the MAPK/ERK pathway enhances OPC differentiation.^[^ ^19^ ^]^ Besides, the interplay between MAPK signaling cascades and the cell cycle machinery, especially cyclin D1 and cyclin‐dependent kinases, is well established during the mouse embryos development.^[^ ^20^ ^]^ To test the correlation between calcium signaling and CDK6/cyclin D1 after *Itpr2* loss in OPCs, Western blot assay was used to examine the activation of MAPK canonical pathway in OPC cultures. Consistent with the results in qRT‐PCR assay, CDK6 protein level showed a 1.8‐fold increase in *Itpr2* cKO OPCs (Figure 4F,G), in agreement with more proliferating OPCs in *Itpr2* cKO pups (Figure 2C,D). Besides, the phosphorylated cRaf and ERK1/2 showed a 1.7 and 2.2‐fold increase in *Itpr2* cKO OPC cultures, respectively, with stable level of total cRaf and total ERK1/2 (Figure 4F,H,I). We noticed that although the expression of cyclin D1 was not changed after *Itpr2* ablation, the phospho‐cyclin D1 level showed 3.97‐fold increase in *Itpr2* cKO cells (Figure 4F,J). These results may explain the increased EdU incorporation and delayed differentiation after *Itpr2* ablation.

### 2.5 Antagonist Against ERK Pathway Rescued the Hypomyelination Phenotype and Depressive‐Like Behaviors in *Itpr2* cKO Mice

**Figure 5:**
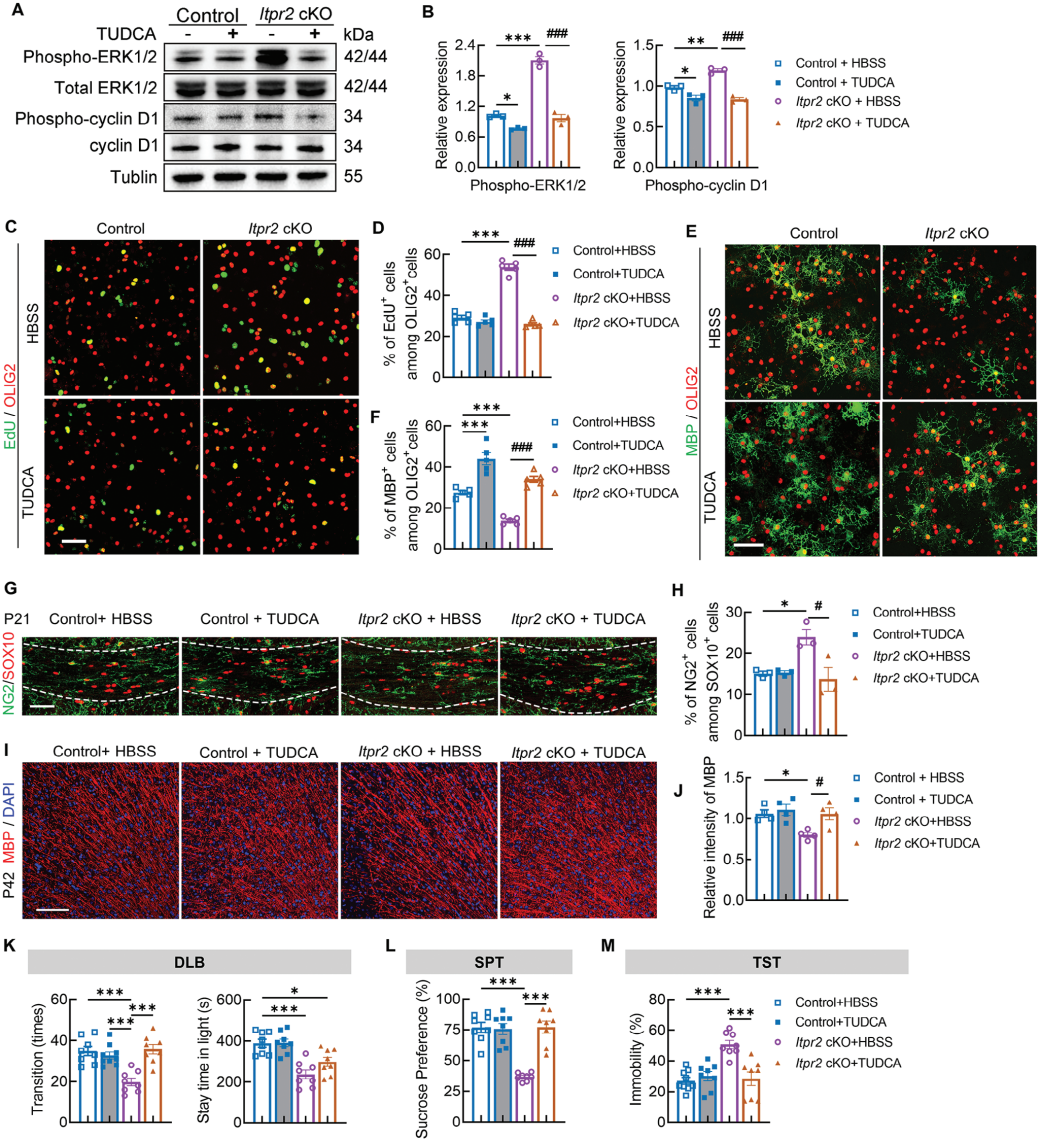
*Itpr2* ablation activated cyclin D1 through MAPK/ERK pathway. A) Western blot analysis for Phospho‐cyclin D1 (p‐cyclin D1) and Phospho‐ERK1/2 (pERK) in either control or *Itpr2* cKO OPC treated with TUDCA (200 mm) for 24 h. Tubulin was used as loading control. B) Relative p‐cyclin D1 and pERK1/2 level in each group as indicated. (*n* = 3 replicates in each group). Data presents Mean ± SEM; One‐Way ANOVA and Tukey's multiple comparisons test were used to compare the mean of each with other columns. *, compared with control + HBSS (*, *p* <0.05; **, *p* <0.01; ***, *p* <0.001); **#**, *Itpr2* cKO+TUDCA versus *Itpr2* cKO+HBSS (**###**, *p* <0.001). C) Immunostaining of EdU and OLIG2 for proliferating OPC cultures in control and *Itpr2* cKO cells treated with HBSS or TUDCA. Bar = 50 µm. D) Quantification the percentage of EdU^+^ cells among OLIG2^+^ cells in control and *Itpr2* cKO OPCs treated with HBSS or TUDCA (*n* = 5 individual cultured cell coverslips in each group). Data presents Mean ± SEM; One‐Way ANOVA and Tukey's multiple comparisons test. *, compared with control+HBSS (***, *p* <0.001); **##**, *Itpr2* cKO+TUDCA versus *Itpr2* cKO+HBSS (*p* <0.01). E) Immunostaining of MBP and OLIG2 for differentiating OL cultures in control and *Itpr2* cKO cells treated with HBSS or TUDCA. Bar = 50 µm. F) Quantification the percentage of MBP^+^ cells among OLIG2^+^ cells in control and *Itpr2* cKO cells treated with HBSS or TUDCA (*n* = 5 individual cultured cell coverslips in each group). Data presents Mean ± SEM; One‐Way ANOVA and Tukey's multiple comparisons test. *, compared with control+HBSS (***, *p* <0.001); **##**, *Itpr2* cKO+TUDCA versus *Itpr2* cKO+HBSS (*p* <0.01). G) Immunostaining of NG2 and SOX10 in cc from P21 control and *Itpr2* cKO mice treated with HBSS or TUDCA. Bar = 100 µm. H) Quantification the percentage of NG2^+^ cells among SOX10^+^ cells in cc from control and *Itpr2* cKO mice treated with HBSS or TUDCA (*n* = 3 mice per group). Data presents Mean ± SEM; One‐Way ANOVA and Tukey's multiple comparisons; *, compared with control+HBSS (*p* <0.05); **##**, *Itpr2* cKO+TUDCA versus *Itpr2* cKO+HBSS (*p* <0.01). I) Immunostaining of MBP in cortex and cc from P21 control and *Itpr2* cKO mice treated with HBSS or TUDCA. Bar = 100 µm. J) Quantification of relative MBP intensity in cortex and cc from control and *Itpr2* cKO mice treated with HBSS or TUDCA (*n* = 3 mice per group). Data presents Mean ± SEM; One‐Way ANOVA and Tukey's multiple comparisons. *, compared with control+HBSS (*, *p* <0.05; **, *p* <0.01; ***, *p* <0.001); **##**, *Itpr2* cKO+TUDCA versus *Itpr2* cKO+HBSS (**#**, *p* <0.05; **##,** *p* <0.01). K) Transition times (left) and stay time in light (right) in DLB from four groups as indicated (*n* = 8 mice in each group). Data presents Mean ± SEM; One‐Way ANOVA and Tukey's multiple comparisons; *, *p* < 0.05; ***, *p* <0.001. L) Percentage of sucrose consumption volume in SPT from four groups as indicated (*n* = 8 mice in each group). Data presents Mean ± SEM; One‐Way ANOVA and Tukey's multiple comparisons; ***, *p* <0.001. M) Percentage of immobility time in TST from four groups as indicated (*n* = 8 mice in each group). Data presents Mean ± SEM; One‐Way ANOVA and Tukey's multiple comparisons; ***, *p* <0.001.

To verify the involvement of the MAPK/ERK pathway in ITPR2‐Ca^2+^ regulated cyclin D1 activation and OPC differentiation, we investigated the effect of inhibiting ERK activation by Tauroursodeoxycholate (TUDCA) application. TUDCA has been shown to inhibit both the proliferation and migration of vascular smooth muscle cells via the inhibition of ERK.^[^ ^21^ ^]^ As expected, TUDCA (200 µM) significantly decreased the level of phosphorylated ERK1/2 in OPC after 24‐hours incubation (**Figure** **5**A,B; Figure S7A,B, Supporting Information). Quantification the relative immunoblot band intensity revealed reduced levels of phospho‐ERK1/2 in *Itpr2* cKO OPC cultures after TUDCA addition (control+HBSS, 1.01 ± 0.02; control+TUDCA, 0.76 ± 0.01; *Itpr2* cKO+HBSS, 2.10 ± 0.07; *Itpr2* cKO+TUDCA, 0.97± 0.07). Besides, TUDCA treatment normalized phospho‐cyclin D1 level in *Itpr2* cKO OPCs (control+HBSS, 0.98 ± 0.02; control+TUDCA, 0.85 ± 0.03; *Itpr2* cKO+HBSS, 1.19 ± 0.02; *Itpr2* cKO+TUDCA, 0.84 ± 0.02), which is one of MAPK downstream targets (Figure 5B).

This result prompted us to further test the effect of TUDCA in enhancing OPC differentiation. We noticed that TUDAC application twice in 24‐h significantly decreased the percentage of EdU^+^ cells in OPC culture from *Itpr2* cKO mice, as revealed by immunostaining (Figure 5C,D). Besides, TUDCA treatment four times, with 12‐h intervals each time, increased the percentage of MBP^+^ cells in OL cultures from both control and *Itpr2* cKO mice (Figure 5E,F). Similarly, oral gavage application of TUDCA in pups from P3‐P15 can greatly reduce the percentage of NG2^+^ OPCs at P21 and enhance MBP expression at P42 in both control and *Itpr2* cKO mice (Figure 5G–J).

Additionally, we tested the anxiety and depressive behaviors in *Itpr2* cKO mice after the TUDAC application as described above. In the dark/light box transition test, TUDCA treatment increased the transition times of P35 *Itpr2* cKO mice, compared to the vehicle treatment (Figure 5K). In the sucrose preference test, TUDCA treatment significantly enhanced sucrose consumption in P38 *Itpr2* cKO mice, compared to vehicle treatment (Figure 5L). *Itpr2* mutant at P45 treated with TUDCA revealed decreased immobility time than vehicle‐treated mutant in tail resuspension test (Figure 5M), indicating the alleviated anxiety/depressive mood in these mice. Besides, we noticed that TUDCA application in control mice did not show significant effects on their behavioral performance.

Together, these data confirmed that ITPR2 loss in OLs induces the activation of cyclin D1 through the MAPK/ERK cascade, which delays their progression into the differentiation stage and interrupts OL‐associated behaviors. Interestingly, MAPK pathway activation is usually correlated with higher calcium signaling activity; therefore, it remains unclear how the cytoplasm calcium level changed after *Itpr2* deletion.

### 2.6 Increased Resting [Ca^2+^]_i_ Level and Membrane Calcium Channel Activity after *Itpr2* Ablation in OLs

**Figure 6:**
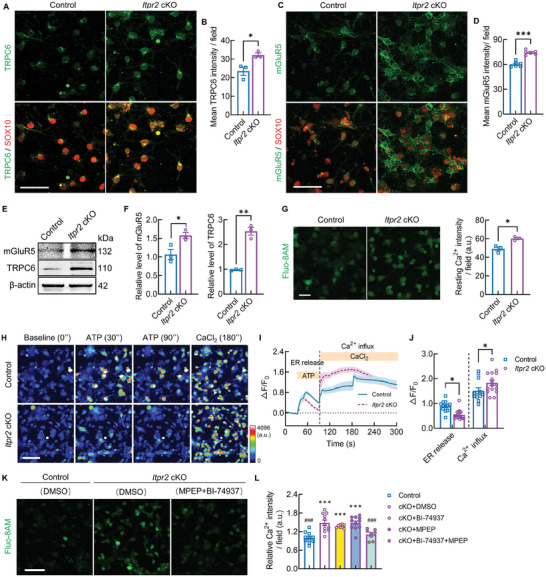
Increased resting [Ca^2+^]_i_ level and calcium channel activity in *Itpr2* cKO OPCs. A) Immunostaining of TRPC6 and SOX10 in OPC from control and cKO mice. Bar = 50 µm. B) Quantification of TRPC6 intensity in control and cKO OPCs (*n* = 3 coverslips from at least 3 mice each group). Data presents Mean ± SEM; Two tail unpaired *t*‐test; *, *p* <0.05. C) Immunostaining of mGluR5 and SOX10 in OPC from control and cKO mice. Bar = 50 µm. D) Quantification of mGluR5 intensity in control and cKO OPCs (*n* = 6 coverslips from at least 3 mice each group). Data presents Mean ± SEM; Two tail unpaired *t* test; ***, *p* <0.001. E) Western blot analysis showed increased levels of TRPC6 and mGluR5 in OPCs from cKO mice. F) Quantification of mGluR5 and TRPC6 level in control and cKO OPCs (*n* = 3 replicates in each group). Data presents Mean ± SEM; Two tail unpaired *t* test; *, *p* <0.05; ***, *p* <0.001. G) Imaging and quantification of resting Ca^2+^ in control and cKO OPC cultures labelled with Fluo‐8 AM (*n* = 3 independent cultured coverslips). Bar = 50 µm. Data presents Mean ± SEM; Two tail unpaired *t*‐test; *, *p* <0.05. H) Representative serial heatmap of calcium imaging for living OPC cultures from control and cKO mice. OPCs loaded with Fluo‐8 AM were analyzed for Ca^2+^ transients following the addition of ATP calcium imaging as described in materials and methods. ATP‐induced Ca^2+^ release from ER. After initial analysis in [Ca^2+^]‐free buffer, the Ca^2+^ influx from the extracellular milieu was determined upon addition of 2 mm CaCl_2_ in the presence of ATP. Bar = 50 µm. I) Representative trace of Ca^2+^ release and influx indicated by Fluo‐8 fluoresces intensity in OPCs following addition of ATP and CaCl_2_ sequentially (*n* = 12 traces for control and cKO group from 3 individual cell cultures, respectively). J) Quantification the mean intensity of Ca^2+^ signal traces of 12 images from the OPC culture slides. Data presents Mean ± SEM; Two‐way ANOVA and Šídák's multiple comparisons test were applied for compare the Fluo‐8 AM intensity of cKO OPC in Ca^2+^ release or Ca^2+^ influx with control OPC. *, *p* <0.05. K) Representative imaging of resting Ca^2+^ labeled with Fluo‐8 AM in control and cKO OPC cultures treated with DMSO or MPEP+BI‐74937. Bar = 50 µm. L) Quantification of relative resting Ca^2+^ intensity in control and cKO OPC cultures treated with DMSO, BI‐74937, MPEP, and MPEP+BI‐74937. (*n* = 11 coverslips in control, cKO+DMSO, and cKO+MPEP group; *n* = 8 coverslips in cKO+ BI‐74937 and cKO+ MPEP+BI‐74937 group). Data presents Mean ± SEM; One‐Way ANOVA and Dunnett's multiple comparisons test; ***, *p* <0.001 (compared to control), ###, *p* <0.001 (compared to cKO+DMSO).

From RNA‐Seq data, we noticed that a group of cytomembrane‐located calcium channels is upregulated in OPCs from *Itpr2* cKO mice. In OPC cultures from *Itpr2* cKO mice, immunostaining showed increased TRPC6 and mGluR5 expression compared to control cultures (**Figure** **6**A,D). In addition, protein extractions from OPC cultures revealed higher levels of TRPC6 and mGluR5 in *Itpr2* cKO group by Western blot assay (Figure 6E,F). Notably, both mGluR5 and TRPC6 showed the highest mRNA level in newly formed oligodendrocytes as ITPR2 (Figure S7C,D, Supporting Information, OL1), which is consistent with the previous study.^[^ ^22^ ^]^ These data indicate that *Itpr2* ablation may induce the compensatory elevation of other calcium channels in cell membrane, which then influence the resting [Ca^2+^]_i_ in OPCs.^[^ ^23^ ^]^

To test the conjecture, we first used cytoplasm Ca^2+^ indicator Fluo‐8 AM to evaluate the level of free cytosolic Ca^2+^ concentration ([Ca^2+^]_i_) in OPC cultures. As shown in Figure 6G, the resting [Ca^2+^]_i_ intensity in cKO OPCs was higher than that in control OPCs, suggesting that loss of *Itpr2* elicits a rise in basal [Ca^2+^]_i_, which may influence Ca^2+^ signals according to similar research.^[^ ^24^ ^]^ In addition, we measured the calcium influx ability in the absence of intercellular calcium to verify the activity of increased calcium channels on cell membrane of *Itpr2* cKO OPCs. ATP application was used to evacuate Ca^2+^ from ER store, which showed significantly decreased Ca^2+^ oscillating in cKO group (Figure 6H,I). Then the cells were subjected to 2 mm CaCl_2_ in extracellular milieu to measure capacitive Ca^2+^ entry through the membrane calcium channels. We found that calcium influx wave was much higher in cKO group compared with control OPCs (Figure 6H–J), consistent with the increased level of TRPC6 on the cell membrane.

Finally, we measured the resting [Ca^2+^]_i_ in OPC cultures from *Itpr2* cKO mice that were treated with TRPC6 or/and mGluR5 antagonists. According to previous studies,^[^ ^25^ ^]^ BI‐74937 (500 nm) and MPEP (20 µm) were used to block TRPC6 and mGluR5, respectively. Two hours after antagonist addition, cell cultures were subjected to Fluo‐8 AM incubation and confocal microscope recording revealed significantly reduced resting [Ca^2+^]_i_ in *Itpr2* cKO cultures that treated with combination of BI‐74937 and MPEP (Figure 6K,L). The comparable fluorescence intensity in BI‐74937 or MPEP individually treated *Itpr2* cKO OPC cultures indicate that multiple calcium channels are influenced after ITPR2 ablation and blocking single one is not enough to restore the normal level of [Ca^2+^]_i_ in OPCs.

These observations confirmed our speculations that *Itpr2* ablation may induce the elevated activity of membrane calcium channels in OPCs and result in higher resting [Ca^2+^]_i_ levels. Therefore, CDK6 expression and cyclin D1 activation level mediated by the MAPK pathway are enhanced. These changes finally interfere with cell cycle progression and delay OPC differentiation, which may cause depressive and anxious‐like behaviors in the mice.

## 3 Discussion

Although a wealth of evidence shows that extrinsic signals to OPCs through numerous factors may converge on the regulation of [Ca^2+^]_i_ via multiple calcium channels, whether or how Ca^2+^ signaling actually affects OPC and oligodendrocyte biology is relatively unclear. Our results showed that ITPR2‐mediated calcium homeostasis is required for proper development of OPCs, and conditional ablation of *Itpr2* in OLs leads to anxiety and depressive‐like behaviors in the animals. Detailed mechanism analysis revealed that although calcium release from the endoplasmic reticulum was repressed, *Itpr2* deletion in OPCs caused higher cytoplasmic [Ca^2+^]_i_ level in these cells, possibly through compensatory Ca^2+^ influx from other membrane calcium channels. The resulting hyperactivity of calcium signaling may activate MAPK/ERK pathway, which promotes the expression of genes involved in cell cycle regulation and inhibits OPC differentiation. Our results reveal the novel role of ITPR2 in oligodendrocyte development and function, therefore providing possible therapeutic strategies for demyelination‐associated disorders.

### 3.1 Depletion of *Itpr2* in Oligodendrocytes‐Induces Anxiety and Depressive‐Like Behavior in the Mice

Depression is a serious mental disorder involving loss of interest or persistent sadness. Recently, multiple studies have suggested the involvement of glial cells, especially oligodendrocytes, in the pathogenesis of depression.^[^ ^3^, ^4^, ^26^ ^]^ These studies indicate the abnormal myelin integrity both in patients and in the mice model. In current study, two lines of oligodendrocyte‐specific *Itpr2* knockout mice were generated, and both *Itpr2* cKO and *Itpr2* iKO mice showed impaired myelination and anxiety/depressive‐like behaviors. These results suggested a novel mechanism in regulating OL lineage progression and further strengthened the contribution of dysmyelination in depression. Aside from the cell autonomous effect of ITPR2 in OLs, we noticed that Cao and colleagues had shown that lack of ITPR2 induced deficiency in astrocytic ATP release, causing depressive‐like behaviors in *Itpr2* null mice.^[^ ^27^ ^]^ In addition, Gao's research found that astrocyte‐specific ITPR2 conditional knockout mice displayed autism‐like behaviors, such as atypical social interaction and repetitive behavior^[^ ^28^ ^]^ and these animals showed no change in anxiety/depressive behaviors, with standard motor and sensory ability, as well as normal learning and memory functions.^[^ ^29^ ^]^ Our findings extended the function of ITPR2 beyond astrocytes, and suggested the significance of ITPR2 in oligodendrocytes involved neural function. Whether there exist non‐cell autonomous effects in oligodendrocytes, e.g. ATP secretion failure is open to be investigated.

### 3.2 *Itpr2* Deficiency Impairs Ca^2+^ Release from ER and Disrupts Developmental OPC Transition into Pre‐OLs

Many studies suggest that Ca^2+^‐mediated signal transduction in oligodendrocytes plays an essential role in myelin development.^[^ ^30^ ^]^ Spontaneous or neuronal activity dependent Ca^2+^ oscillations in OPCs control proliferation, differentiation, and myelin sheath morphological changes.^[^ ^6^, ^31^ ^]^ For example, Ca^2+^ release mediated by ryanodine receptor on the endoplasmic reticulum has been validated to be important for regulating oligodendrocyte maturation.^[^ ^32^ ^]^ As another way of releasing [Ca^2+^]_i_ from ER, the underlying mechanism of inositol 1,4,5‐trisphosphate receptor, ITPR2, remains elusive. ITPR2 was identified as a distinct postmitotic oligodendrocyte subclass marker by scRNA‐seq for the brain,^[^ ^10^ ^]^ and we speculate that the dynamic high level of ITPR2 in these cells may indicate its stage specific role during OL lineage progression.

Mei and colleagues have revealed that *Itpr2* deficiency may reduce the percentage of OLs that prefer to myelinate large‐diameter axons in the CNS.^[^ ^33^ ^]^ In current study, we noticed that TUNEL assay did not reveal significant changes in apoptotic OLs from *Itpr2* iKO mice and therefore ruled out the possible effect of *Itpr2* deletion on reducing OLs numbers by cell death. However, EdU incorporation assay in OPC cultures showed an increased proportion of proliferating OPCs after *Itpr2* ablation, consistent with the increased percentage of PDGFRα^+^ or NG2^+^ OPCs in *Itpr2* iKO mice brain. Interestingly, in vivo EdU incorporation revealed less mature OLs in EdU positive cells in *Itpr2* iKO mice, indicating the essential role of ITPR2 in controlling OPC transition into differentiating stage. Together with the results that oligodendrocyte pool in *Itpr2* iKO mice did not expand with the increased proliferating OPCs, we believe that ITPR2 mediated calcium homeostasis is involved in cell cycle regulation.

### 3.3 Deletion of *Itpr2* Impairs Cell Cycle Machinery Through Activation of MAPK/ERK Pathway

Consistent with the above results, transcriptome analysis identified dysregulated genes involved in cell cycle transition, myelination, and calcium ion homeostasis in *Itpr2*‐deleted OPCs. We noticed that key players in cell cycle progression, such as CDK6, were upregulated in *Itpr2‐*depleted OPCs, which is part of the G1 cell cycle checkpoint that allows the cell to enter the S‐phase.^[^ ^34^ ^]^ Therefore, we believe that the higher proportion of EdU^+^ OPCs in *Itpr2* cKO mice may result from the increased CDK6 expression, which accelerates the progression of cell cycle into S‐phase.

It has been known that Cyclin D1 enhances the transition of cells through the G1 phase of the cell cycle by binding to and activating CDK4 or CDK6, which were downstream targets of the MAPK/ERK signaling pathway in most cases.^[^ ^35^ ^]^ We then confirmed that canonical MAPK/ERK pathway, including phosphorylation of cRaf and ERK1/2, was activated in *Itpr2* cKO OPCs, which may cause the upregulation of CDK6 and phospho‐cyclin D1. Besides, the application of ERK1/2 inhibitor TUDCA not only promoted OPC maturation and myelination in *Itpr2* cKO mice both in vitro and in vivo, but also alleviated depressive like behaviors in *Itpr2* mutant. Therefore, we believe that the activated ERK pathway in OPCs from *Itpr2* mutant may modulate the expression of cell cycle regulators, such as CDK6 and cyclin D1, that facilitate G1‐S phase transition. The abnormal cell cycle progression may finally result in failed developmental transition from OPC to pre‐OL and inhibit OPC differentiation.

### 3.4 The Compensatory Effect after *Itpr2* Ablation Involves Elevated Resting [Ca^2+^]_i_ in OLs

MAPK/ERK pathway activation has been identified as a downstream factor for [Ca^2+^]_i_ elevation.^[^ ^36^ ^]^ It is known that multiple calcium channels on cell membrane and organellar advances the efficiency of Ca^2+^ signaling in OPC, and inter‐organellar exchange determines the overall concentration of cytosolic Ca^2+^. Considering the higher resting [Ca^2+^]_i_ in OPC cultures from *Itpr2* cKO mice, we believe that once the calcium release from ER is prohibited, compensatory mechanism may be started as response.

To be consistent with this speculation, we detected hyperactivation of other plasma membrane calcium channels, such as TRPC6 and mGluR5, in *Itpr2‐*depleted OPCs. TRP channels allow entry of Ca^2+^ and Mg^2+^ better than Na^+^, and several TRP channels have been suggested to be involved in OPC development.^[^ ^37^ ^]^ The level of TRP cation channel subfamily C (TRPC) is developmentally regulated in OPCs, and extracellular cation entry through TRPC1 is an essential component in the mechanism of OPC proliferation.^[^ ^38^ ^]^ Besides, activation of the TRP channel subfamily V member 4 (TRPV4) significantly increased the proliferation of OPCs.^[^ ^39^ ^]^ In our study, both Ca^2+^ evacuation test and antagonist blocking test in OPC cultures revealed the contribution of these membrane calcium channels in elevating resting [Ca^2+^]_i_ after ITPR2 loss, therefore suggesting the essential function of ITPR2 in maintaining OPC calcium homeostasis_._

In summary, we propose that ITPR2 is crucial for OPC development and the underlying molecular machinery changes following *Itpr2* depletion will finally interfere with OPC proliferation/differentiation progression and result in abnormal behaviors in the animal. Thus, our findings show the significance of calcium homeostasis in oligodendrocyte biology and open a new window for comprehending the potential of OLs in the onset and therapeutic strategies for depression.

## 4 Experimental Section

### Animal Experiments

All animal experiments were performed following the National Institutes of Health Guidelines on the Use of Laboratory Animals and approved by the Air Force Medical University Committee on Animal Care. All mice in this study were housed in an animal room with a controlled environment of 22–24 °C, 45–65% humidity, and a 12 h light/dark cycle. The Olig1Cre mice were gifted by Dr. Q. Richard Lu.^[^ ^15b^ ^]^ The NG2Cre^ER^ mice^[^ ^40^ ^]^ were from Jackson lab (Strain #:0 08533). *Itpr2* ^fl/fl^ mice^[^ ^41^ ^]^ were provided by the RIKEN BRC (RBRC10293) through the National Bio‐Resource Project of the MEXT/AMED, Japan. *Itpr2* ^fl/fl^ mice were crossed with Olig1Cre or NG2Cre^ER^ mice to generate *Itpr2* cKO or *Itpr2* iKO mice. To induce NG2Cre expression in *Itpr2* iKO mice, tamoxifen (Sigma, Cat #T5648) was dissolved in corn oil (Sigma, C8267) and injected intraperitoneally at 3 mg^−1^ 40 g (body weight) per day. Olig1Cre or NG2Cre^ER^ mice were used as control as the previous study.^[^ ^7^ ^]^

### Additional Materials and Methods

Detailed methods are included in the Supporting Information. Cell culture, molecular, immunohistology, and behavioral studies were performed on age‐matched male and female mice. No significant differences were observed when comparing genotypes based on sexes, therefore, male and female data were pooled (additional information in Supporting Information). Statistical analyses were performed using the Prism v.9.0 (GraphPad Software, Inc.) and listed in supplementary materials.

## Conflict of Interest

The authors declare no conflict of interest.

## Supporting information

Supporting Information

## Data Availability

The data that support the findings of this study are available from the corresponding author upon reasonable request.
