# Supplementary material for: ITPR2 Mediated Calcium Homeostasis in Oligodendrocytes is Essential for Myelination and Involved in Depressive‐Like Behavior in Adolescent Mice
Source: Adv Sci (Weinh). 2024 Mar 13;11(20):2306498. doi: 10.1002/advs.202306498 (PMC11132048; doi:10.1002/advs.202306498)
Supplement: Supplementary file 1 — Supporting Information [file ADVS-11-2306498-s001.pdf]

ADVANCED SCIENCE Open Access

Supporting Information

for *Adv. Sci.*, DOI 10.1002/advs.202306498

ITPR2 Mediated Calcium Homeostasis in Oligodendrocytes is Essential for Myelination and Involved in Depressive-Like Behavior in Adolescent Mice

Ming Zhang, Na Zhi, Jiaxiang Feng, Yingqi Liu, Meixia Zhang, Dingxi Liu, Jie Yuan, Yuhao Dong, Sufang Jiang, Junye Ge, Shengxi Wu and Xianghui Zhao**

Supporting information:

ITPR2 mediated calcium homeostasis in oligodendrocytes is essential for myelination and involved in depressive-like behavior in adolescent mice

Ming Zhang[1†], Na Zhi[1,2,†], Jiaxiang Feng[1], Yingqi Liu[1], Meixia Zhang[3], Dingxi Liu[4], Jie Yuan[1,2], Yuhao Dong[1], Sufang Jiang[1], Junye Ge[1], Shengxi Wu[1,*], Xianghui Zhao[1,*]

Supplemental materials and methods
Figure S1
Figure S2
Figure S3
Figure S4
Figure S5
Figure S6
Figure S7
Table S1
Supplementary references

Supplemental materials and methods

Mouse oligodendrocyte precursor cell primary culture

Mouse OPCs were isolated from the cortices of pups at P6 cortices as described previously[1]. Cortical tissues were digested with trypsin (Gibco, 25200072) and dispersed into single cells. The cell suspension was subjected into immunopanning dishes: RAN-2 (ATCC #TIB-119, 1:5 hybridoma supernatant), anti-GalC (1:5 hybridoma supernatant), and O4 (1:5 hybridoma supernatant) sequentially at room temperature[2]. $O4^+$ $GalC^−$ OPCs was released from the final panning dish with trypsin. OPCs were cultured in Sato medium[3] containing the following elements: DMEM/F12 (Gibco, 11330-032), 4 mM L-glutamine (Sigma, G8540), 1 mM sodium pyruvate (Sigma, P8574), 0.1% BSA (Sigma, A9647), 50 mg/ml Apo-transferrin (Sigma, T2252), 5 mg/ml insulin (Sigma, I6634), 30 nM sodium selenite (Sigma, S5261), 10 nM D-biotin (Sigma, H1759) and 10 nM hydrocortisone (Sigma, H0888). PDGF-AA (10 ng/ml, Peprotech, 100-13A) and bFGF (10 ng/ml, Peprotech, 100-18B) were added into the Sato medium for OPC proliferation, and 10 ng/mL CNTF (Peprotech, 450-13), 5 mg/mL NAC (Sigma, A8199) and 40 ng/mL T3 (Sigma, T2877) for oligodendrocytes differentiation.

Ca^{2+} imaging for OPC cultures

Calcium indicator Fluo-8 AM (Abcam, Cat #ab142773) was used to measure the cytoplasmic Ca^{2+} concentrations. OPC was preloaded with Fluo-8 AM (2 μM) by incubation at 37°C for 20 min and washed three times with HBSS to remove extracellular Fluo-8 AM. After washing, cells were transferred into calcium-free ACSF

containing (mM): NaCl 125, KCl 3.0, $MgCl_2$ 2.0, $MgSO_4$ 2.0, NaH_2PO_4 1.25, $NaHCO_3$ 26, glucose 20, HEPES 10, and EGTA 10 (pH = 7.4). A recording chamber on the stage of an inverted confocal microscope (Olympus FV3000, Japan) equipped with phase-contrast optics was used. Fluo-8 AM was excited with a 488 nm laser, and emitted light was collected at 515 nm.

To measure the resting Ca^{2+}, a series of imaging were captured every 1s intervals, for 10 s in total. The mean intensity of Fluo-8 AM were analyzed using the CellSense Dimension software (Olympus).

To measure ER calcium release, a series of imaging were collected every 500 ms, at 500 ms intervals, for 180 s in total. To induce calcium release,100 μM ATP (Sigma, A1852) was added into ACSF at the 30s after starting recording.

To measure Ca^{2+} influx from extracellular milieu through calcium channels on cell membrane, the OPC cultures were first incubated in Ca^{2+} free ACSF and added 100 μM ATP at 30s as described above to empty Ca^{2+} in ER. This was followed by subjecting cells to external 2 mM $CaCl_2$ at 90s to measure capacitive Ca^{2+} entry from the extracellular milieu. Serial images were recorded continuously for 300s.

Lateral ventricle microinjection and Ca^{2+} imaging for brain slices

To measure calcium release in oligodendrocytes from brain slices, rAAV2/9-CAG-FLEX-GCaMP7s (1 μl, PT1421, BrainVTA Co., Ltd) was injected into lateral ventricle of anesthetized P1 mice using 10 μl Hamilton Microliter Syringes (701 N) equipped with insulin pen needles (BD microfine plus 4 mm). The injection needle was withdrawn 3 min after the infusion. Fourteen days later, coronal slices (300 μm) were

prepared from Olig1Cre (Control) and *Itpr2* cKO mice as indicated. Animals were anesthetized with pentobarbital; the brain was quickly removed and placed in ice-cold oxygenated modified calcium-free ACSF. Then acute brain slices containing anterior cingulate cortex region were prepared using a VT-1200S vibratome (Leica, Germany) at the cutting speed of 0.1 mm/s. Slices were then incubated at 34 °C in oxygenated ACSF for 30 min before experiments. Oligodendrocytes expressing GCaMP7s were selected for imaging.

For evaluating calcium released from ER, the slides were transferred to calcium-free ACSF as before. A two-photon microscope (Olympus FV1200MPE, Japan) equipped with a 25×, 1.05 NA water-immersion objective was used for slice imaging. GCaMP7s was exited at 920 nm with a Mai Tai Ti: Sapphire laser. During the 180 s recording, ATP (100 μM) was added at 30s. The imaging speed was set at 1s interval with 512 × 512 pixels in each frame.

Time-lapse images of the OPC cultures and brain slices were analyzed using CellSense Dimension software (Olympus) to acquire the fluorescence intensity in the region of interest (ROI) in each frame.

The peak amplitude for OPC and brain slide were calculated using the formula:

$$(\Delta F/F_0) = (F_{max} - F_0)/F_0,$$

where F_{max} was the maximum fluorescence intensity after addition of ATP and F_0 was the average basal fluorescence intensity before ATP application.

Tissue preparation and immunofluorescence staining

Mice were anesthetized with isoflurane and perfused with cold PBS through the left

cardiac ventricle, followed by 4% paraformaldehyde (PFA, Sigma-Aldrich, 158127). Brain was removed and post-fixed in fresh fixative for 2 hours at 4°C. To prepare cryosection, samples were dehydrated in 30% sucrose in PBS for 48 hours at 4°C prior to embedding in an OCT mounting medium. Coronal sections (15 μm) were mounted on glass slides and subjected to immunostaining. Antigen retrieval (Citotest, 188105) was performed before staining, and the samples were blocked in 5% bovine serum albumin with 0.5% Triton X-100 for 2 hrs. Samples were incubated with primary antibodies overnight at RT, followed by washing with PBS three times and staining with fluorescein-conjugated secondary antibodies for 2 hours at room temperature in the dark. After PBS washing, sections were incubated in DAPI solution (28718-90-3, Solarbio) for nuclear staining and mounted with 50% glycerin in PBS. Staining results were visualized and recorded using Olympus FV3000 confocal microscopy.

The list of antibodies used was as follows:

Primary antibodies: ITPR2 (Santa Cruz sc-398434, 1:100), OLIG2 (Millipore AB9610, 1:500), SOX10 (Santa Cruz 17342, 1:50), PDGFRα (Abcam ab61219, 1:500), NG2 (Cspg4) (Millipore AB5320, 1:500), CC1 (Oncogene Research OP80, 1:200), MBP (Abcam ab7349, 1:500), Ki67 (Abcam ab16667, 1:500), TRPC6 (Proteintech 18236-1-AP, 1:100), mGluR5 (Abcam ab76316, 1:200), NEFL2 (Oasis biofarm OB-MMS032, 1:500), NeuN (Oasis biofarm OB-PRT013, 1:500), GFAP (Genetex GTX108711, 1:500), O4 (gift from Mengsheng Qiu, 1:50).

Secondary antibodies: Donkey anti rabbit Alexa Fluor 488 (Invitrogen A21206, 1:1000), Donkey anti rabbit Alexa Fluor 594 (Invitrogen A21207, 1:1000), Donkey anti

rabbit Alexa Fluor 647 (Invitrogen A31573, 1:1000), Donkey anti guinea pig Alexa Fluor 488 (Invitrogen A11076, 1:1000), Donkey anti mouse Alexa Fluor 594 (Invitrogen A21203, 1:1000), Donkey anti rat Alexa Fluor 594 (Invitrogen A-21209, 1:1000).

Transmission electron microscope

Mice were perfused with saline and then with fixative buffer containing: 2% glutaraldehyde, 2% paraformaldehyde in 0.1 M sodium cacodylate buffer (pH 7.4). Next day, 100 μm slides were prepared with vibratome and further fixed for 24hr. The slides were incubated in 1% osmium tetroxide for 1.5 hr and then dehydrated in graded alcohols and acetone before being subjected to a standard protocol for epoxy resin embedding. 1 μm ultrathin sections were cut onto copper grids and stained with uranyl acetate before being examined with a JEM-1230 electron microscope (JEOL LTD, Tokyo, Japan), which is equipped with a CCD camera and its application software (832 SC1000, Gatan, Warrendale, PA). Myelin analysis was performed double-blind using adobe photoshop. The analysis of myelin thickness was performed at a 25,000× amplification.

Black Gold II staining

Black gold staining was performed according to the protocol (AG105, Sigma-Aldrich). Coronal cryosections (50 μm) were incubated in 0.3% Black-Gold II solution at 60–65 °C for ~12 min and the intensity of staining was monitored under microscope. Then the slides were rinsed for 2 min in ddH_2O and transferred to 1% sodium thiosulfate

solution for 3 min. After rinsing in ddH_2O, the sections were air dried on a slide warmer, immersed in xylene for 2 min and mounted with neutral resins.

Immunocytochemistry and EdU (5-ethynyl-2'-deoxyuridine) labeling

For immunocytochemistry, coverslips were fixed with 4% PFA for 15 min and washed with PBS for 3 times. Samples were then blocked in 3% bovine serum albumin + 0.3% Triton X-100 for 1 hr and incubated with primary antibodies 2hrs at RT. Similar procedures were performed as above for secondary antibody labeling.

For EdU staining of brain slides, mice were intraperitoneal injected with 25mg/kg EdU (Beyotime, C0071L) at P6, and executed 48 hrs later. For cultured OPC cells, 10 μM EdU were added into culture medium 4~6 hrs before fixation. EdU Staining was performed following the manual. Briefly, slides were fixed with 4%PFA for 15 min, and permeabilized with 0.3% Triton X-100 for 10 min. Then 100 μl EdU reaction buffer were added to the slides and incubated at room temperature for 30 min. Following staining protocols were performed as described.

TUNEL assay

TUNEL assay for apoptotic cells were performed according to DeadEnd™ Fluorometric TUNEL System (Promega, G2350) kit instructions. The frozen section slides were immersed in PBS for 5 min, and fixed in 4% PFA for 15 min. After washing twice in PBS, the samples were incubated with 20 μg/ml Proteinase K at room temperature for 8–10 minutes. Slides were immersed in PBS for 5 min and fixed again for 5 min. After equilibration for 5–10 minutes, 50 μl of TdT reaction mix was added

on the slices and plastic coverslips were used to ensure even distribution of the reaction mix. The samples were incubated at 37°C for 1hr and the reaction was stopped by 2× SSC incubation. Finally, slices were washed three times in PBS and counterstain with DAPI.

For TUNEL/OLIG2 double staining, OLIG2 immunostaining were applied after the TUNEL labeling to reveal apoptotic cells in OPC.

Behavioral procedures

Adolescent mice (5–6 weeks) were used for behavioral tests in our study. Two groups of mice were tested respectively, starting from the least to the most stressful experiments. Rotarod test was used to examine the motor coordination in rodents and OFT, DLB and EPM test were used to assess anxiety-related behaviors. TST, SPT and FST were used to examine the depressive like behaviors. The mice were placed in the behavioral room at least 2 hours before the test. The apparatus was cleaned with 70% ethanol and wiped with paper towels between each mouse. All data were analyzed by the experimenters who were blinded to the treatments.

Rotarod test

The rotarod test were used to access the muscle strength and motor coordination ability of the mice [4]. The device consists of a rotating rod for acceleration and four horizonal cylinders to divide each mouse. In the adaptive phase, mice were placed on the rotating rod at a constant speed of 20 rpm for 5 min. The training was conducted twice a day for 3 days until mice can keep moving for 5 minutes without dropping. In the testing phase,

the rotating rod accelerates from 4 rpm to 30 rpm in 5 minutes. A total of three measurements for one animal were conducted in one day, with each interval no less than 1 hr. The three fall-off time of each mouse was recorded and averaged.

Open Field Test (OFT)

Mice were placed into a square arena (50 × 50 × 40 cm) and allowed to move freely for 10 min, moderately illuminated with a light intensity of 130 lx. All behaviors were recorded by a video camera above the box and analyzed using a video tracking system Smart 3.0 software (Panlab, Harvard Apparatus). The anxiety level of each mouse was evaluated by their activities in the arena's central region (25%) as previously reported [5].

Elevated Plus Maze (EPM)

The EPM apparatus consists of a "+"-shaped maze elevated above the floor, with two oppositely positioned closed arms, two oppositely positioned open arms, and a center area. Mice were placed in the center area to explore the maze freely for 10 min. Their behavior is recorded using a camera. The preference for being in open arms over closed arms (percentage of time spent in the open arms or percentage of distance traveled in the open arms) is calculated to measure anxiety level as reported in previous study [6].

Dark Light Box (DLB) transition test

This test is used to detect activity in disorders related to generalized anxiety and is used to complement the OFT results [7]. The task is based on the innate aversion of rodents to brightly illuminated areas and on the spontaneous exploratory behavior in response

to a novel environment and light. The apparatus consists of a dual-compartment box with free access between them. The dark compartment is made of black Plexiglas and covered with a lid. The other box is exposed and is brightly illuminated by an overhead lamp. Each animal was placed in the center of the illuminated compartment, facing the gate to the dark compartment, and their time spent in the light compartment was recorded for 5 min.

Tail Suspension Test (TST)

The tail-suspension test is common in assessing depression-related behaviors. Mice were suspended by 1/3 position of their tails with tape to prevent escape or hold onto nearby surfaces. Their struggling and immobility behavior were recorded for 8 min and their performance during 2-8 min were used for analysis as in previous study [8].

Sucrose Preference Test (SPT)

As described in previous studies [9], individually housed mice were first habituated to two bottles of water for 24 hrs, followed by two bottles of 1% sucrose for 24 hrs. Mice were deprived of water and food for 24 hrs before measurement. In the sucrose-preference measurement session, mice were given access to a two-bottle choice of water or 1% sucrose solution for 24 hrs. Bottles containing water and sucrose were weighed every 12 hrs. The position of the bottles was interchanged (left to right, right to left) after weight measurement to ensure that the mice did not develop a side bias. Sucrose preference was calculated as the percentage of sucrose consumption among total consumption volume.

Forced swim test (FST)

Mice were placed in a clear Plexiglas cylinder (25 cm high; 10cm in diameter) filled with 24 °C water to a depth of 15 cm. A camera positioned directly in front of the cylinder was used to record the behavior of each mouse for 6 min. The duration of immobility during 2-6 min was recorded as in previous study [10], which was defined as no movement of the limb or body except those caused by respiration.

RNA extraction and qRT-PCR

Total RNAs were purified from OPC cultures using TRIzol reagent (Invitrogen 15596026) according to the manufacturer's instructions. For qRT-PCR, RNA was transcribed to cDNA with the PrimeScript™ RT reagent Kit (Perfect Real Time, Takara RR037A). Reactions were performed with LightCycler® RNA Master SYBR Green I (Roche 03064760001) and quantified by CFX96 Touch Real-Time PCR Detection System (Bio-Rad). Relative gene expression was calculated by Bio-Rad CFX Manager and normalized to internal reference GAPDH. Primer sequences were listed in Supplementary Table1.

RNA seq

Total RNA extraction from control and *Itpr2* cKO OPC cultures was performed using TRIzol. RNA quality was checked with an Agilent 2100 bioanalyzer. The cDNA libraries were constructed using the TruSeq RNA Library Prep Kit (Illumina, San Diego, CA, USA), and the libraries were sequenced on an Illumina HiSeq 2500 system, with paired end read length of ~150 bp. The expressions of transcripts were quantified as

reads per kilobase of exon model per million mapped reads (RPKM). The DEGs were identified using Cuffdiff with p value ≤ 0.05 and further screened by RPKM $|\log_2\text{fold}| \geq 1$. The enrichment analysis was performed and plotted with the GSEA 4.3.2 software (https://www.gsea-msigdb.org/gsea/downloads.jsp). Heatmap was plotted by https://www.bioinformatics.com.cn (last accessed on 10 July 2023), an online platform for data analysis and visualization. The RNA-seq data has been deposited in the NCBI BioProject database (accession number PRJNA952894).

Western Blot analysis

For Western blot assay, OPC cultures were lysed using RIPA buffer and after centrifugation, supernatant protein concentrations were measured by BCA Protein Detection Kit (Pierce, 23225). Equal amounts of proteins (25 μg) were separated by 10% SDS-PAGE and transferred to a Hybond PVDF membrane (Roche, 03010040001). The membranes were blocked with 15% non-fat dry milk in TBS and incubated with the primary antibody overnight. Target proteins were detected by secondary antibodies conjugated with horseradish peroxidase (Abbkine, 1:5000) and signals were developed with chemiluminescence ECL system (Zeta LIFE). Primary antibodies used were ERK1/2 (CST 9212S, 1:1000), Phospho-ERK1/2 (Proteintech 28733-1, 1:3000), cRaf (Boster BM4108, 1:800), Phospho-cRaf (Boster BM4780, 1:800), CDK6 (Proteintech 19117-1, 1:2500), Cyclin D1 (CUSABIO CSB-PA004811KA01HU, 1:500), Phospho-Cyclin D1 (Beyotime AG1215, 1:1000), and β-Tubulin (Abbkine A01030, 1:10000). The bands were quantified with ImageJ V4.5.1 software and the intensity were normalized to Tubulin level as relative fold change against control.

TUDCA application *in vitro* and *in vivo*

To block MAPK/ERK pathway in OPC cultures, 200 μM TUDCA (APE Bio, HY-19696) was added twice every 12 hrs after plating. Cells were collected for Western blot assay 24hrs after the first addition of TUDCA, or induced to differentiation for another 48hrs and collected for immunostaining assay. Hank's balanced salt solution (HBSS) solvent was used as control. For *in vivo* application of TUDCA, mice pups were treated with TUDCA by daily oral gavage (200 mg / kg body weight) in *Itpr2* cKO mice from postnatal day 3 to 15. The dose was referred from previous studies[11] and HBSS-treated *Itpr2* cKO mice from the same litters were compared at postnatal day 21 for OPC differentiation and myelination by immunostaining. In order to test the effect of TUDCA application in alleviating anxiety and depressive like behaviors in *Itpr2* cKO mice, dark light box transition test, sucrose preference test and tail suspension test were applied sequentially in P35, P38 and P45 mice as mentioned above.

Application of antagonists against TRPC6 and mGluR5 in OPC cultures

To confirm the hyperactivity of membrane calcium channels after *Itpr2* ablation in oligodendrocytes, antagonists against TRPC6 (BI-74937, 500nM, MedChemExpress HY-111925) or mGluR5 (MPEP, 50 μM, MedChemExpress HY-14609) were applied in OPC cultures individually or simultaneously. Purified OPC cultures from *Itpr2* cKO mice were treated twice with antagonists with 12hrs interval, and examined for resting $[Ca^{2+}]_i$ by calcium imaging 2hrs after last antagonist addition.

Statistical Analysis

All the statistical analysis was performed using the Prism v.9.0 (GraphPad Software, Inc.). The normality test was performed by the Shapiro–Wilk test and the homogeneity of variance test was performed by Levene's test. All data met normality and homogeneity of variance were compared using a Two-tailed unpaired *t*-test or One Way ANOVA. Data sets that were normal distribution but not homogeneity of variance was compared using a Two-tailed unpaired separate variance estimation *t*-test. Data didn't meet normal distribution were analyzed with Mann-Whitney U test. Numerical values were described using Mean ± SEM. Significance is denoted as *p<0.05, **p<0.01 or ***p<0.001 in the figures.

Supplementary Figures

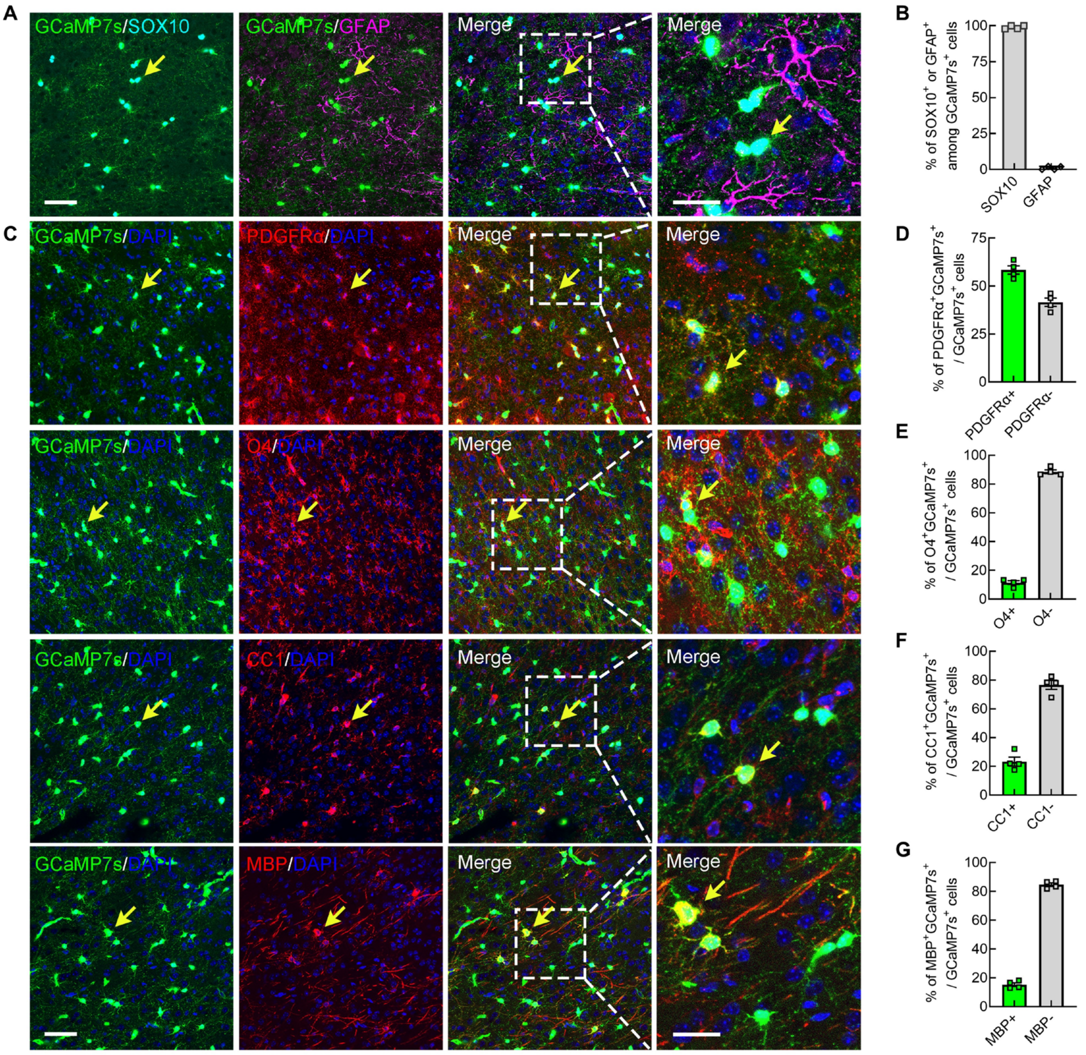

Figure S1. Immunostaining the brain slices from P14 Olig1Cre mice expressing GCaMP7s-GFP revealed the specificity of GFP in oligodendrocytes.

A. Confocal microscopy images show GCaMP7s$^+$ cells expressing pan oligodendrocyte lineage marker SOX10 (cyan) rather than astrocyte marker GFAP (magenta). Higher magnification of the boxed area was enlarged on the right. Scale bars, 50 µm (left) and 25µm (right).

B. Percentage of SOX10$^+$ and GFAP$^+$ cells among GCaMP7s$^+$ cells. (n= 4 mice for each group). Data presents Mean ± SEM.

C. Images showing the expression of GCaMP7s in different subset of oligodendrocytes. PDGFRα (OPC), O4 (immature OL), CC1 and MBP (mature

and myelinating OL). Higher magnification of the boxed area was enlarged on the right. The yellow arrow indicates the co-labelled cells. Scale bars, 50 μm (left) and 25 μm (right).

D-G. Quantification the percentage of PDGFRα, O4, CC1 and MBP^+ cells among $GCaMP7s^+$ cells, respectively. (n= 4 mice for each group). Data presents Mean ± SEM.

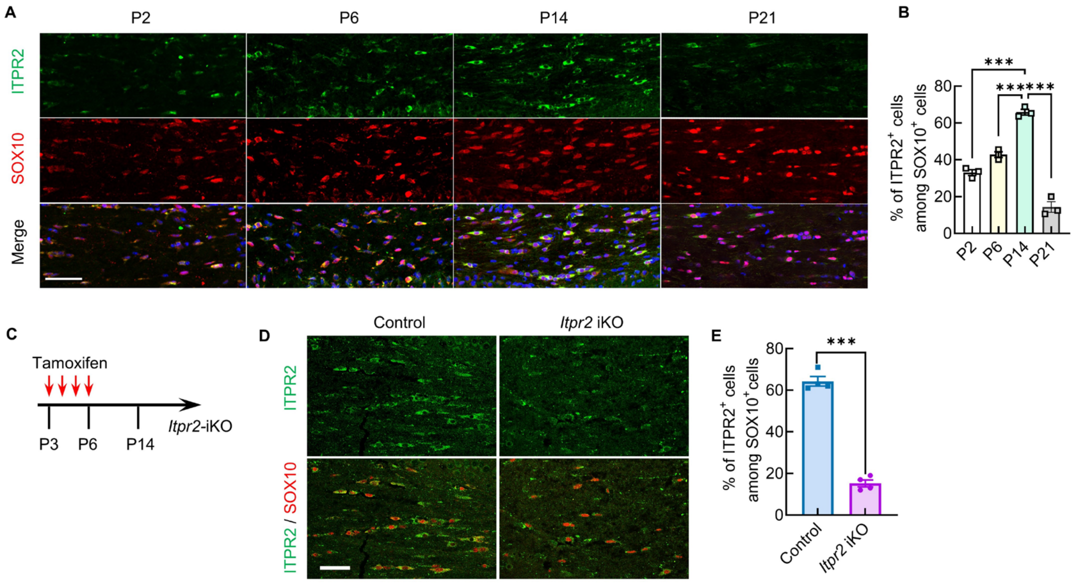

Figure S2. Stage dependent expression of ITPR2 in oligodendrocytes during brain development and construction of *Itpr2* iKO mice.

A. Immunofluorescence for ITPR2 and SOX10 in corpus callosum from wild type mice at different developmental stage as indicated. Scale bar, 100 µm.

B. Percentage of ITPR2$^+$ cells among SOX10$^+$ cells at different developmental stage (n= 3 mice for each group). Data presents Mean ± SEM; One Way ANOVA, ***, $p < 0.001$.

C. Schematic diagram of Tamoxifen administration in *Itpr2* $^{flox/flox}$; NG2CreER mice (*Itpr2* OPC-iKO).

D. Representative immunostaining for ITPR2 expression in postnatal day 14 (P14) corpus callosum from control and *Itpr2* iKO mice. Upper images show only ITPR2 staining, lower images show ITPR2 (green) and SOX10 (red) staining. Scale bar, 50 µm.

E. Percentage of ITPR2$^+$ cells among SOX10$^+$ oligodendrocytes in P14 corpus callosum of control and *Itpr2* iKO mice. Data were Means ± SEM. (n=4 animals in each group). Two-tailed unpaired *t*-test; ***, $p < 0.001$.

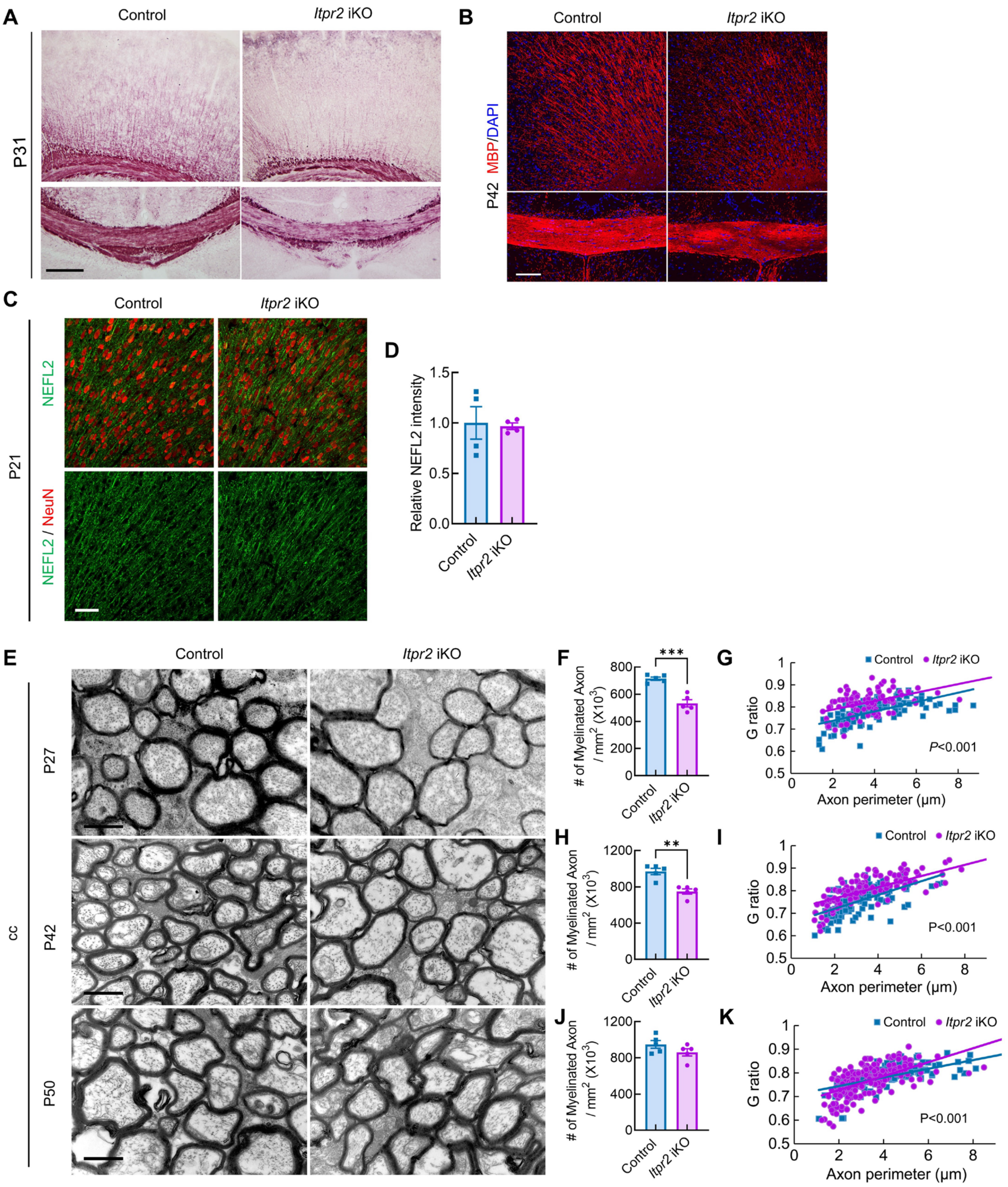

Figure S3. ITPR2 ablation affects myelination but not axon degeneration.

A. Representative images of black gold staining for myelin in cortex and corpus callosum of control and *Itpr2* iKO mice at P31. Bar = 200 µm.

B. Representative images of MBP immunostaining in cortex and corpus callosum from control and *Itpr2* iKO mice at P42. Bar = 100 µm.

C. Immunostaining of NEFL2 and NeuN in cortex of P21 control and iKO mice, Bar = 50 µm.

D. Quantification of relative fluorescence intensity for NEFL2 in cortex of control

and *Itpr2* iKO mice at P21 (n=4 animals for each group). Data presents Mean ± SEM; Two tail unpaired *t* test.

E. Representative electron micrographs of P27, P42 and P50 corpus callosum from control and *Itpr2* iKO mice. Bar = 1 μm.

F. Quantification of the number of myelinated axons in defined areas from the corpus callosum of P27 control and *Itpr2* iKO mice. Data were Means ± SEM (n=5 mice for each group). Two tail unpaired *t* test; ***, $p < 0.001$.

G. G ratios vs. axonal perimeters for P27 corpus callosum in control and *Itpr2* iKO mice. (n =125 myelinated axon counts/animal, from five animals/genotype). Friedman M test, $p_{(\text{between group})} < 0.001$.

H. Quantification of the number of myelinated axons in defined areas from the corpus callosum of P42 control and *Itpr2* iKO mice. Data were Means ± SEM (n=5 mice for each group). Two tail unpaired *t* test; *, $p < 0.05$.

I. G ratios vs. axonal perimeters for P42 corpus callosum in control and *Itpr2* iKO mice. (n =120 myelinated axon counts/animal, from five animals/genotype). Friedman M test, $p_{(\text{between group})} < 0.001$.

J. Quantification of the number of myelinated axons in defined areas from the corpus callosum of P50 control and *Itpr2* iKO mice. Data were Means ± SEM (n=5 mice for each group). Two tail unpaired *t* test.

K. G ratios vs. axonal perimeters for P50 corpus callosum in control and *Itpr2* iKO mice. (n >150 myelinated axon counts/animal, from five animals/genotype). Friedman M test, $p_{(\text{between group})} <0.001$.

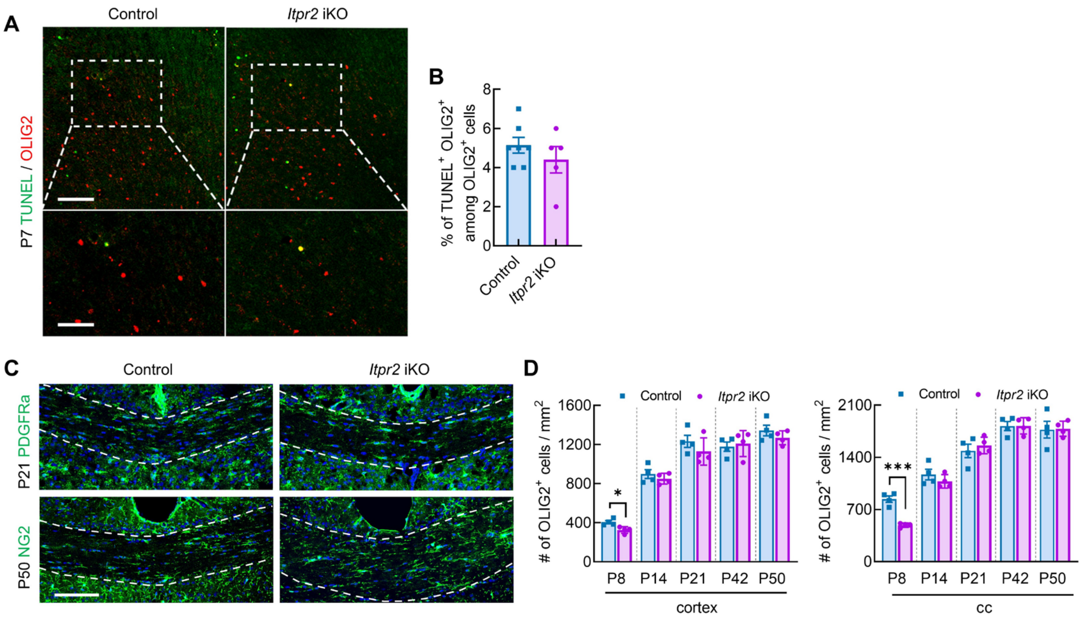

Figure S4. ITPR2 ablation affects density of OPC but not oligodendrocyte apoptosis.

A. Immunostaining of OLIG2 co-labelled with TUNEL from cortex of P7 control and *Itpr2* iKO mice, Bar = 100 μm. Enlarged images for white box area were shown in the lower panel, Bar = 50 μm.

B. Percentage of $TUNEL^+$ cells among $OLIG2^+$ cells (n=7 slides from 3 control mice and n=5 slides from 3 iKO mice). Data presents Mean ± SEM; Two tail unpaired *t* test.

C. Representative images of immunostaining for PDGFRα and NG2 in corpus callosum from control and iKO mice at P21 and P50, respectively. Bar = 100 μm.

D. Quantification of OLIG2 density in cortex (left) and cc (right) from control and *Itpr2* iKO mice at P8, P14, P21, P42 and P50. Data presents Mean ± SEM. Two-way ANOVA, Šídák's multiple comparisons test were used to compare the mean of *Itpr2* iKO with control mice in each time, *, $p < 0.05$, ***, $p < 0.001$.

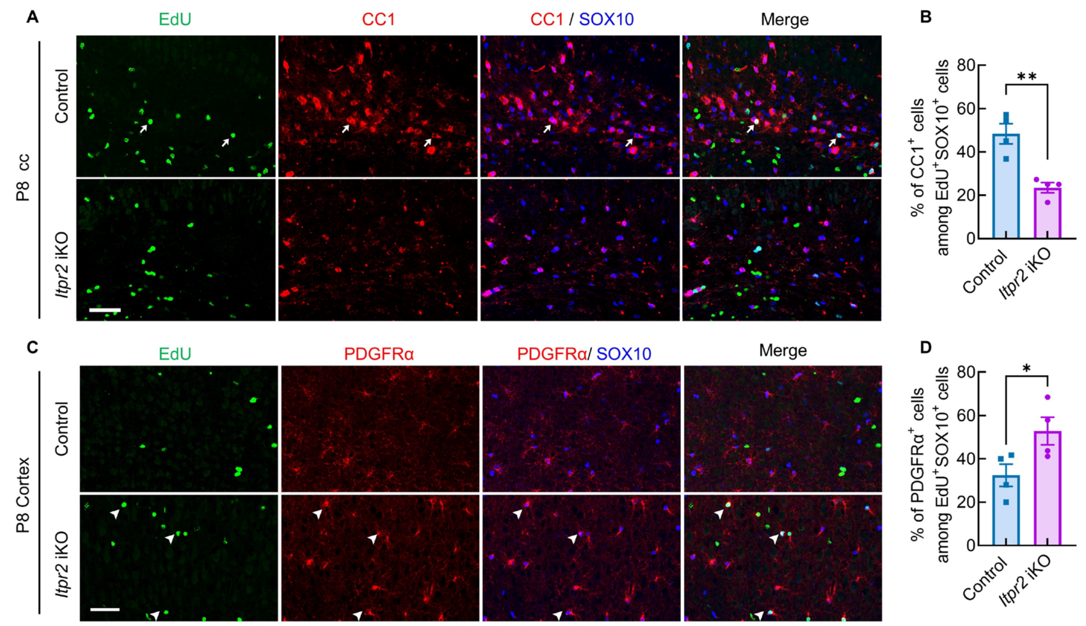

Figure S5. ITPR2 ablation affect OPC transition to pre-OL stage.

A. Immunostaining of CC1 co-labelled with SOX10 in cc at P8 after 2 days EdU administration from control and iKO mice, Bar = 50 μm. Arrows indicated $CC1^+$ OLs differentiated from EdU labeled OPCs.

B. Percentage of $CC1^+$ cells among EdU^+OLIG2^+ cells (n=4 mice each group). Data presents Mean ± SEM; Two tail unpaired *t* test, **, $p < 0.01$.

C. Immunostaining of PDGFRα co-labelled with SOX10 in cortex at P8 after 2 days EdU administration from control and iKO mice, Bar = 50 μm. Arrowheads indicated $PDGFR\alpha^+$ OPCs that were EdU positive.

D. Percentage of $PDGFR\alpha^+$ cells among EdU^+ $SOX10^+$ cells (n=4 mice each group). Data presents Mean ± SEM; Two tail unpaired *t* test, *, $p < 0.05$.

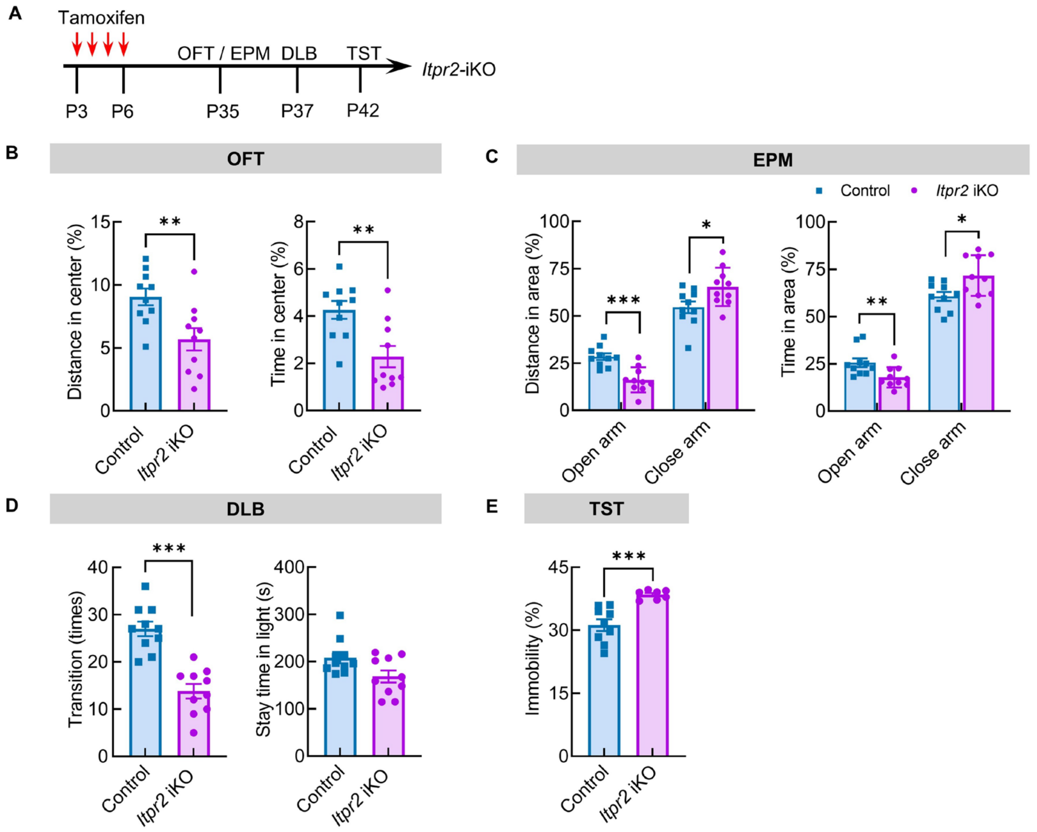

Figure S6. Increased depression/anxiety level in *Itpr2* iKO mice.

A. Schematic diagram of Tamoxifen injection and behavioral test plan for *Itpr2* iKO mice. OFT, open field test; EPM, elevated plus maze; DLB, dark light box; TST, tail suspension test.

B. Percentage of traveling distance (left) and time (right) in center zone of the open field. Data presents Mean ± SEM (n=10 animals for each group). Two tail unpaired *t* test; **, $p < 0.01$.

C. Percentage of traveling distance (left) and time (right) in open and close arm of EPM. Data presents Mean ± SEM (n=10 animals for each group). Two tail unpaired *t* test; *, $p < 0.05$; ***, $p < 0.001$.

D. The transition times across the dark/light box (left) and stay time in light box (right) in DLB transition test. Data presents Mean ± SEM (n=10 animals for each group). Two tail unpaired *t* test; ***, $p < 0.001$.

E. Percentage of immobility time in TST. Data presents Mean ± SEM (n=9 control mice and n=7 iKO mice). Two tail unpaired *t* test; ***, $p < 0.001$.

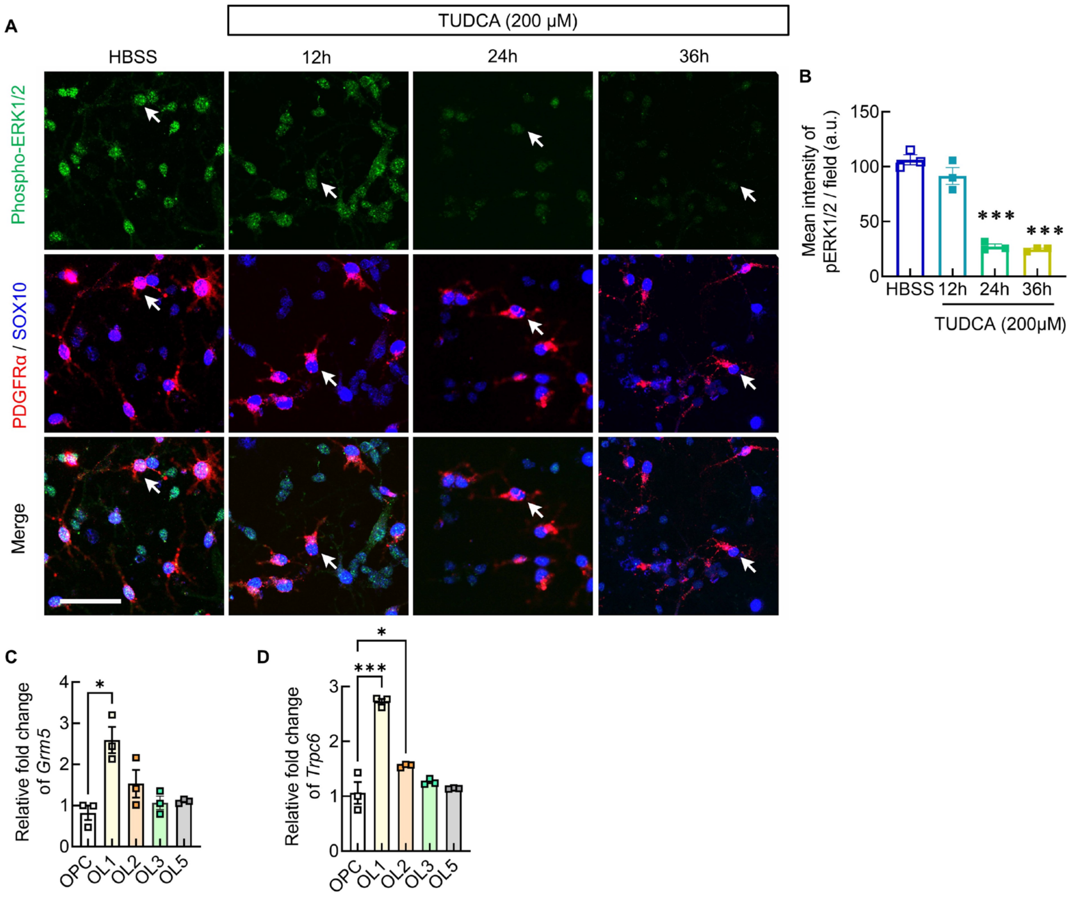

Figure S7. TUDCA inhibits phosphorylation of MAPK(ERK1/2) in OPC cultures.

A. Immunostaining of phospho-ERK1/2 (pERK1/2), PDGFRa and SOX10 in OPC cultures after TUDCA treatment for 12, 24, and 36hrs. Bar = 50 μm. Arrows indicate the intensity of pERK1/2 after incubated with HBSS or TUDCA (200 μM).

B. Quantification of pERK1/2 fluorescence intensity in OPC (n= 3 independent cell culture in each time point). Data presents Mean ± SEM; One Way ANOVA, ***, p <0.001 compared with HBSS group.

C. Relative expression of *Grm5* at different differentiation stage (n= 3 replicates for each group). Data presents Mean ± SEM; Dunnett's multiple comparisons test, *, p<0.05 (compared with OPC).

D. Relative expression of *Trpc6* at different differentiation stage (n= 3 replicates for each group). Data presents Mean ± SEM; One Way ANOVA and Tukey's multiple comparisons test (compared with OPC), *, p<0.05; ***, p<0.001.

Table S1. Primer sequences for qRT-PCR assay of target genes.

Gene	Forward primer	Reverse primer
β-actin	GGCTGTATTCCCCTCCATCG	CCAGTTGGTAACAATGCCATGT
Itpr2	CCTCGCCTACCACATCACC	TCACCACTCTCACTATGTCGT
Hes5	AGTCCCAAGGAGAAAAACCGA	GCTGTGTTTCAGGTAGCTGAC
Fgfr3	GCCTGCGTGCTAGTGTTCT	TACCATCCTTAGCCCAGACCG
Nkx6.2	AAGTCTGCCCCGTCTCAAC	AAGTCTGCCCCGTCTCAAC
Sox10	ACACCTTGGGACACGGTTTTC	TAGGTCTTGTTCCTCGGCCAT
Mbp	GCAGCCAGCACCACTCTTGA	CAGCCGAGGTCCCATTGTTC
Fgf	AGAGTCCAAGAGTAAAAGCAGC	CTTCCGAGGTTCAGCTCTCC
Cdk6	GGCGTACCCACAGAAACCATA	AGGTAAGGGCCATCTGAAAACT
Bmp2	GGGACCCGCTGTCTTCTAGT	TCAACTCAAATTCGCTGAGGAC
Trpc6	TGCTAGAAGAGTGTCATTCCCT	CCTCCACAATCCGTACATAACC
Grm3	CTGGAGGCCATGTTGTTTGC	CATCCACTTTAGTCAACGATGCT
Grm5	CCCAGCACAAGTCGGAAATAG	TGTCTGGTTGGGGTTCTCCTT

Supplementary references

[1] B. Emery, J. C. Dugas, *Cold Spring Harb Protoc* **2013**, *2013* (9), 854, https://doi.org/10.1101/pdb.prot073973.

[2] a) B. Ranscht, P. A. Clapshaw, J. Price, M. Noble, W. Seifert, *Proc Natl Acad Sci U S A* **1982**, *79* (8), 2709, https://doi.org/10.1073/pnas.79.8.2709; b) I. Sommer, M. Schachner, *Dev Biol* **1981**, *83* (2), 311, https://doi.org/10.1016/0012-1606(81)90477-2.

[3] Y. Chen, V. Balasubramaniyan, J. Peng, E. C. Hurlock, M. Tallquist, J. Li, Q. R. Lu, *Nat Protoc* **2007**, *2* (5), 1044, https://doi.org/10.1038/nprot.2007.149.

[4] J. Nicks, S. Lee, A. Harris, D. J. Falk, A. G. Todd, K. Arredondo, W. A. Dunn, Jr., L. Notterpek, *Neurobiol Dis* **2014**, *70*, 224, https://doi.org/10.1016/j.nbd.2014.06.023.

[5] S. K. Jones, D. M. McCarthy, C. Vied, G. D. Stanwood, C. Schatschneider, P. G. Bhide, *Proc Natl Acad Sci U S A* **2022**, *119* (49), e2213120119, https://doi.org/10.1073/pnas.2213120119.

[6] Y. Wang, Y. Su, G. Yu, X. Wang, X. Chen, B. Yu, Y. Cheng, R. Li, J. C. Saez, C. Yi, L. Xiao, J. Niu, *Adv Sci (Weinh)* **2021**, *8* (16), e2101181, https://doi.org/10.1002/advs.202101181.

[7] A. Ramos, *Trends Pharmacol Sci* **2008**, *29* (10), 493, https://doi.org/S0165-6147(08)00165-X [pii]
10.1016/j.tips.2008.07.005.

[8] A. Can, D. T. Dao, C. E. Terrillion, S. C. Piantadosi, S. Bhat, T. D. Gould, *J Vis Exp* **2012**, (59), e3769, https://doi.org/10.3791/3769.

[9] Y. Li, P. Su, Y. Chen, J. Nie, T. F. Yuan, A. H. Wong, F. Liu, *J Clin Invest* **2022**, *132* (8), https://doi.org/10.1172/JCI152187.

[10] N. Jiang, B. Y. Zhang, L. M. Dong, J. W. Lv, C. Lu, Q. Wang, L. X. Fan, H. X. Zhang, R. L. Pan, X. M. Liu, *Phytother Res* **2018**, *32* (6), 1023, https://doi.org/10.1002/ptr.6040.

[11] X. Lu, R. R. Yang, J. L. Zhang, P. Wang, Y. Gong, W. F. Hu, Y. Wu, M. H. Gao, C. Huang, *Fundam Clin Pharmacol* **2018**, *32* (4), 363, https://doi.org/10.1111/fcp.12367.
